# Computational identification of biomarker genes for lung cancer considering treatment and non-treatment studies

**DOI:** 10.1186/s12859-020-3524-8

**Published:** 2020-12-03

**Authors:** Mona Maharjan, Raihanul Bari Tanvir, Kamal Chowdhury, Wenrui Duan, Ananda Mohan Mondal

**Affiliations:** 1 School of Computing and Information Sciences, Florida International University, Miami, FL USA; 2 School of Natural Sciences and Mathematics, Claflin University, Orangeburg, SC USA; 3 Department of Human & Molecular Genetics, Herbert Wertheim College of Medicine, Florida International University, Miami, FL USA

**Keywords:** Bioinformatics, Computational identification of biomarker, Lung cancer biomarkers, Non-treatment studies, Treatment studies

## Abstract

**Background:**

Lung cancer is the number one cancer killer in the world with more than 142,670 deaths estimated in the United States alone in the year 2019. Consequently, there is an overreaching need to identify the key biomarkers for lung cancer. The aim of this study is to computationally identify biomarker genes for lung cancer that can aid in its diagnosis and treatment. The gene expression profiles of two different types of studies, namely non-treatment and treatment, are considered for discovering biomarker genes. In non-treatment studies healthy samples are control and cancer samples are cases. Whereas, in treatment studies, controls are cancer cell lines without treatment and cases are cancer cell lines with treatment.

**Results:**

The Differentially Expressed Genes (DEGs) for lung cancer were isolated from Gene Expression Omnibus (GEO) database using R software tool GEO2R. A total of 407 DEGs (254 upregulated and 153 downregulated) from non-treatment studies and 547 DEGs (133 upregulated and 414 downregulated) from treatment studies were isolated. Two Cytoscape apps, namely, CytoHubba and MCODE, were used for identifying biomarker genes from functional networks developed using DEG genes. This study discovered two distinct sets of biomarker genes – one from non-treatment studies and the other from treatment studies, each set containing 16 genes. Survival analysis results show that most non-treatment biomarker genes have prognostic capability by indicating low-expression groups have higher chance of survival compare to high-expression groups. Whereas, most treatment biomarkers have prognostic capability by indicating high-expression groups have higher chance of survival compare to low-expression groups.

**Conclusion:**

A computational framework is developed to identify biomarker genes for lung cancer using gene expression profiles. Two different types of studies – non-treatment and treatment – are considered for experiment. Most of the biomarker genes from non-treatment studies are part of mitosis and play vital role in DNA repair and cell-cycle regulation. Whereas, most of the biomarker genes from treatment studies are associated to ubiquitination and cellular response to stress. This study discovered a list of biomarkers, which would help experimental scientists to design a lab experiment for further exploration of detail dynamics of lung cancer development.

## Background

Lung cancer is the number one cancer killer in the world with more than 142,670 deaths estimated in the United States alone in the year 2019 [1]. Lung cancer is also the second most common cancer in the world. It is broadly classified into two categories - Non-Small Cell Lung Carcinoma (NSCLC) consisting of 80% of all lung cancer cases and Small Cell Lung Carcinoma (SCLC) recorded in 20% of all lung cancers [2]. The NSCLC is further divided into Adenocarcinoma (40%), Squamous Cell Carcinoma (27%) and large cell carcinoma (8%) [3]. Lung cancer has a low 5-year survival rate of 18% [4]. The low survival rate of lung cancer is due to late diagnosis and relapse of lung cancer after treatment [5]. The late diagnosis plays a significant role in the survival of patients. So, new methods for screening and diagnosis of lung cancer patients which would improve the prognosis need to be developed. The advancement in research and technology has been slowly shifting the focus of lung cancer diagnosis, prognosis and treatment towards understanding the underlying cause of disease progression using protein-protein interaction (PPI) networks, gene co-expression networks and molecular pathways. Though the PPI and co-expression networks are static in nature, these come with rich information about the dynamic processes such as behavior of genetic networks in response to DNA damage [6], prediction of protein subcellular localization [7–12], protein function [13], genetic interaction [14], process of aging [15], and protein network biomarkers [16–22]. The networks are of special interest because the genes do not act alone. They act as a group to achieve a collective goal. In their recent work, Mondal et al. [19] showed that proteins or genes achieved their collective goals by forming clique-like and bipartite graphs, which could be the building blocks for disease initiation and progression. Using these building blocks, Tanvir et al. [20] discovered network modules related to cancers from gene co-expression networks. These literatures suggest that network modules do correlate to specific functions. These motivates us to discover biomarker genes using network-based apps, Cytohubba [23] and MCODE (Molecular Complex Detection) [24], which are two plugins of Cytoscape [25]. This work is the extended version of our previous work [26], which used only three of twelve algorithms available in Cytohubba to discover the biomarker genes. In the present work, all of twelve algorithms in Cytohubba along with another Cytoscape app, MCODE, are used to discover the biomarker genes.

In this study, *first*, DEGs are identified from GEO database using GEO2R tools [27]. *Second*, a functional network is created with these DEG genes using Cytoscape plugin ReactomeFI [28]. *Third*, hub genes are identified from this network using twelve different graph-theoretic algorithms available in Cytohubba, which is another Cytoscape app. *Fourth*, cluster of genes are isolated from the functional network using MCODE, which is also a Cytoscape app. The common genes isolated using Cytohubba and MCODE are considered as the probable biomarker genes for lung cancer.

## Dataset preparation

### Data collection and cleaning

**Fig. 1:**
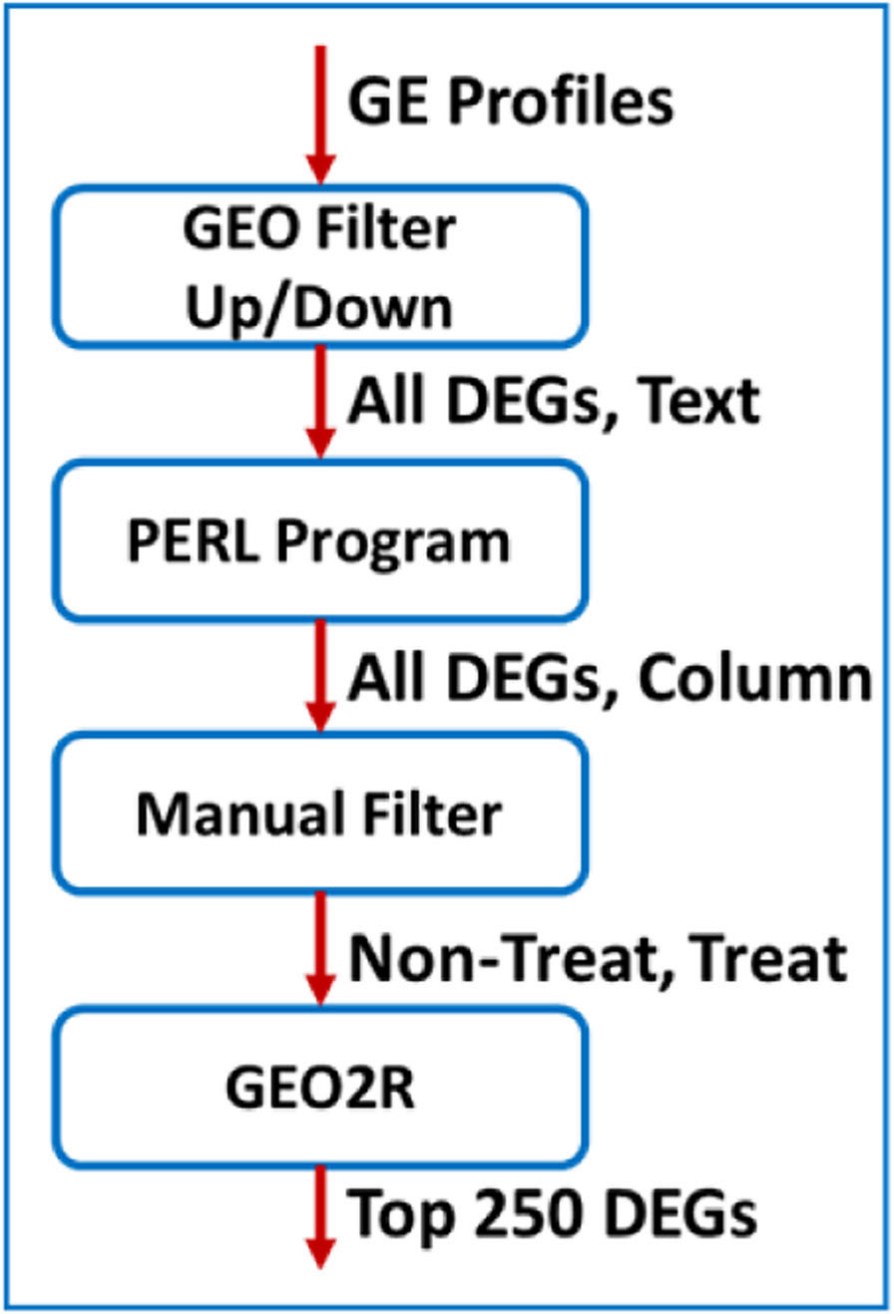
Data cleaning and identification of top 250 DEGs

The gene expression data for lung cancer was collected from GEO database [27]. It provides genome-wide gene expression profiles including DEGs. Figure 1 shows the cleaning and identification of top 250 DEGs that could be probable biomarkers.

Querying GEO database with phrase “lung cancer” retrieved 1,050,133 gene expression (GE) profiles. Using the GEO built-in filter “up/down genes”, the retrieved GE profiles are reduced to 16,876 genes, which are all the DEGs based on GEO filter. The retrieved DEGs were downloaded as a text file, in which each GE profile consists of six lines of record.

A PERL script was used to obtain five important features for each DEG - Gene Symbol, GDS number (study the gene belongs to), organism’s name, and the number of samples used for the study. This intermediate data was stored in column format. From this data, we discovered that the retrieved 16,876 DEGs belong to 27 unique studies.

The scope of the present work is to consider the studies with treatment and non-treatment. Upon using manual filtering - reading the title and abstract of the main publications resulted from these 27 studies - we discovered that only 3 of these studies are non-treatment (GDS1312, GDS4794 and GDS5201) and 2 are treatment (GDS1204 and GDS2499).

In study GDS1204, the lung cancer cell line, A549, is treated with chemotherapeutic drug motexafin gadolinium (MGd) [29]. Three samples are examined at 4, 12, and 24 h following treatment, thus the study consists of 9 control and 9 case samples. In study GDS2499 [30], A549 lung cancer cell lines are treated with two doses of anti-cancer agent sapphyrin PCI-2050 and one dose of transcription inhibitor actinomycin D. The controls are prepared by treating A549 cell lines with mannitol. In each experiment 3 samples are used. This study will provide two sets of DEGs – a) By comparing two doses (applied to 3 samples for each dose, a total of 6 cases) of anti-cancer agent sapphyrin PCI-2050 with 3 control samples; b) By comparing 3 samples with one dose of actinomycin D with 3 control samples.

Two of the non-treatment studies are based on human genome (GDS1312 and GDS4794) and the other is based on mouse genome (GDS5201). In study GDS1312, expression profiling of squamous lung cancer biopsy specimens and paired normal specimens from 5 patients are conducted [31]. The study GDS4794 provides expression profiles of 23 clinical small cell lung cancer (SCLC) samples from patients undergoing pulmonary resection and 42 normal tissue samples from different organs including the lung [32]. In study GDS5201, expression profiles of two mice samples are generated at three genomic variations – normal lung, lung tumor with KrasG12D single mutant, and lung tumor with LSL-KrasG12D double mutant [33]. This study provides one set of DEGs by comparing 4 lung tumor cases with 2 normal lung controls.

Table 1 shows the summary of these 5 datasets including number of case and control. The non-treatment studies are those in which controls are normal samples and cases are cancer patients. Whereas, in treatment studies, both control and case samples are cancer cell lines; samples before treatment are controls and after treatment are cases.

**Table 1:** Summary of expression profile studies for lung cancer. GDS1204 and GDS2499 are treatment studies and the others are non-treatment studies

Study	Data Type	#Samples	#Control	#Case	Cancer Type	Treatment	Reference
GDS1204	Microarray	18	9	9	A549 Cell Line	Yes	[29]
GDS1312	Microarray	10	5	5	Squamous Cell	No	[31]
GDS2499	Microarray	12	3	9	A549 Cell Line	Yes	[30]
GDS4794	Microarray	65	42	23	Small Cell	No	[32]
GDS5201	Microarray	6	2	4	Mouse Model	No	[33]

### Isolating top 250 differentially expressed genes

After identifying the non-treatment and treatment studies, GEO2R, a LIMMA R package in GEO database, was used to isolate top 250 DEGs from each study. The cutoff criteria used were P-value < 0.05 and absolute log_2_ Fold Change (FC) > 1. Benjamini & Hochberg (False Discovery Rate) method was used for adjusting *P*-values. The duplicate DEGs and DEGs with missing symbols were removed from the top 250 DEGs. Finally, a total of 407 DEGs (254 upregulated and 153 downregulated) were discovered from non-treatment studies. Similarly, a total of 547 DEGs (133 upregulated and 414 downregulated) were discovered from treatment studies. There is no common DEGs between non-treatment and treatment studies.

## Methodology

**Fig. 2:**
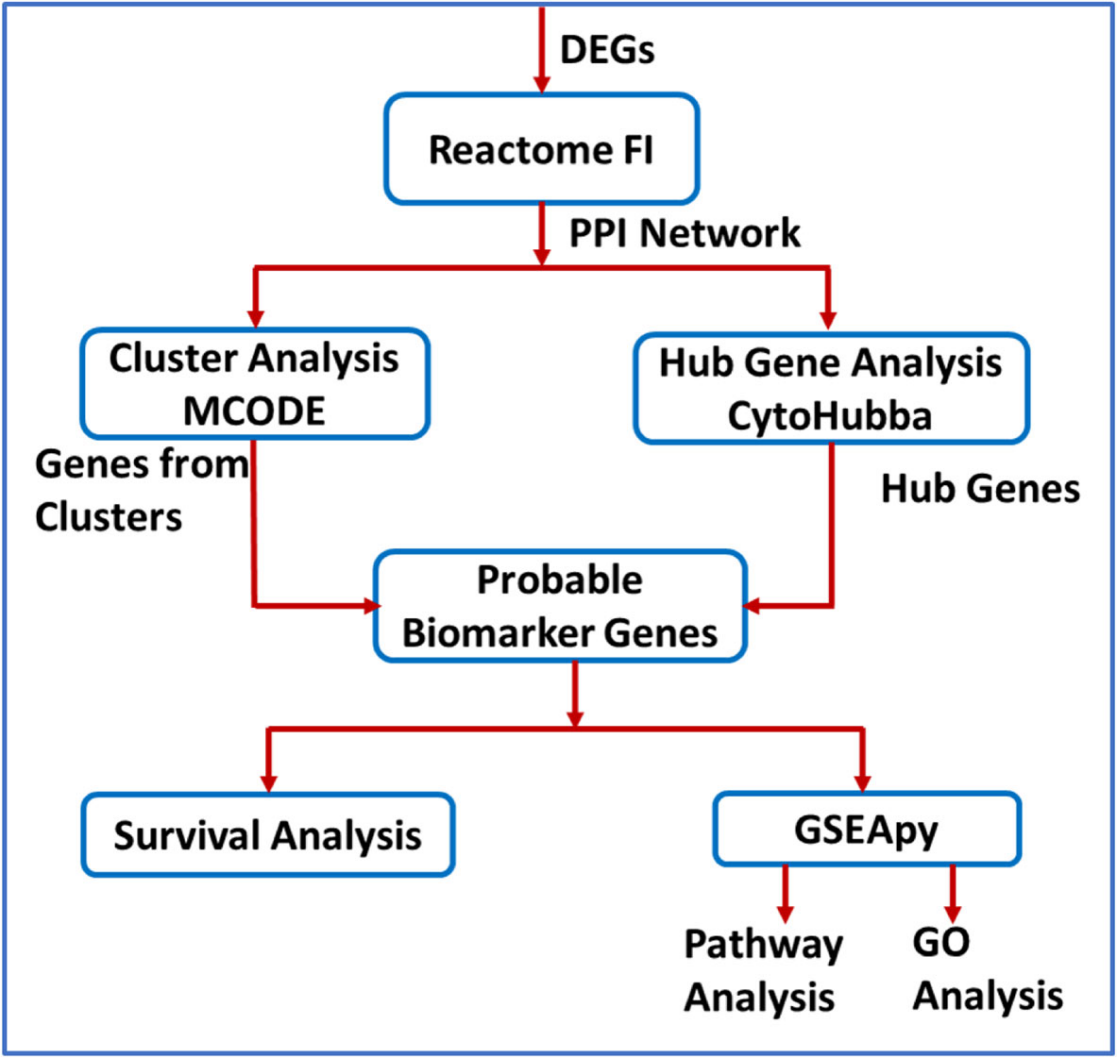
Overview of data analysis methodology

Figure 2 shows the overview of data analysis methodology including i) constructing functional protein network, ii) discovering biomarker genes using Cytohubba and MCODE, iii) functional and pathway analysis of biomarker genes, and iv) survival analysis using biomarker genes.

### Constructing functional protein network

A Cytoscape app, ReactomeFI [28] was used to create two functional protein networks – one consists of non-treatment DEGs and the other consists of treatment DEGs. The resulting two networks have a smaller number of nodes than the original lists, 166 for non-treatment and 260 for treatment, since all DEGs are not functionally related or their functional relation has not yet been discovered. The functional relationship between DEGs was based on Reactome database [34]. These networks were further analyzed to discover probable gene biomarkers using two Cytoscape apps, CytoHubba and MCODE.

### Discovering biomarker genes

The Cytoscape app MCODE [24] was used to find clusters in Non-treatment and Treatment networks constructed above. The default parameters in MCODE (Degree cutoff = 2, Node score cutoff ≥0.2, K-core ≥2 and Max depth from seed = 100) were used for finding the clusters. Another Cytoscape app, CytoHubba [23] was used to find hub genes from the non-treatment and treatment networks. Hub genes are highly connected nodes in the network. The gene network was analyzed using 12 scoring methods available in Cytohubba - betweenness, bottleneck, closeness, clustering coefficient (CC), degree, density of maximum neighborhood component (DMNC), eccentricity (EcC), edge percolated component (EPC), maximal clique centrality (MCC), maximum neighborhood component (MNC), radiality and stress. The top ten genes from each of these methods were isolated. Any gene that is common in at least two scoring methods of Cytohubba and is also present in any of MCODE clusters was considered as a biomarker gene.

### Pathway and GO enrichment analysis

A software package, GSEApy (Gene Set Enrichment Analysis in Python) was used for pathway and GO (Gene Ontology) terms enrichment analysis of biomarker genes. GSEApy is a python wrapper for Enrichr [35] and GSEA (Gene Set Enrichment Analysis) [36]. The analysis was implemented in python 3.7.3 using gseapy package 0.9.5. The adjusted *p*-value < 0.05 and Benjamini & Hochberg correction for multiple testing was used as statistical measure. The pathway analysis was performed using Enrichr ‘KEGG_2019_Human’ library. The enrichr libraries used for GO enrichment are – ‘GO_Biological_Process_2018’, ‘GO_Cellular_Component_2018’ and ‘GO_Molecular_Function_2018’.

### Survival analysis

The survival analysis was performed using online tool Kaplan Meier-Plotter [37]. There are 14 datasets, 12 are collected from GEO database, and the rest two are collected from Cancer Biomedical Informatics Grid (caBIG) and The Cancer Genome Atlas (TCGA). There are a total of 1926 lung cancer samples if the default parameters are chosen. The tool has an option to input either one or multiple genes of interest, then select the factor to split the patients into two groups, such as median (set as default), lower quartile, upper quartile and so on. For the present study, patients with expression value for a gene above the median are included in high-expression group and below the median are included in low-expression group. This system incorporates Kaplan Meier, Logrank method and univariate and multivariate Cox Regression, which are one of the common methods in survival analysis. There are options to restrict the survival analysis based on certain criteria such as histology, stage, grade, gender, surgery success, chemotherapy, radiotherapy.

In the present study, all the samples available (1926) in KM-Plotter was used for survival analysis. However, the number of samples will vary for each gene as only the samples which are relevant to the gene being assessed is used for the analysis [37]. The default parameters were used, meaning, the samples were not restricted based on treatment types or subtypes of cancer. The survival analysis is used to check the prognostic capability of each of the biomarker genes in differentiating between low and high expression groups. For certain genes, more than one probes are available. Without loss of generality, first available probe was used for the present study. FLog.

## Results

### Functionally interacting protein networks

**Fig. 3:**
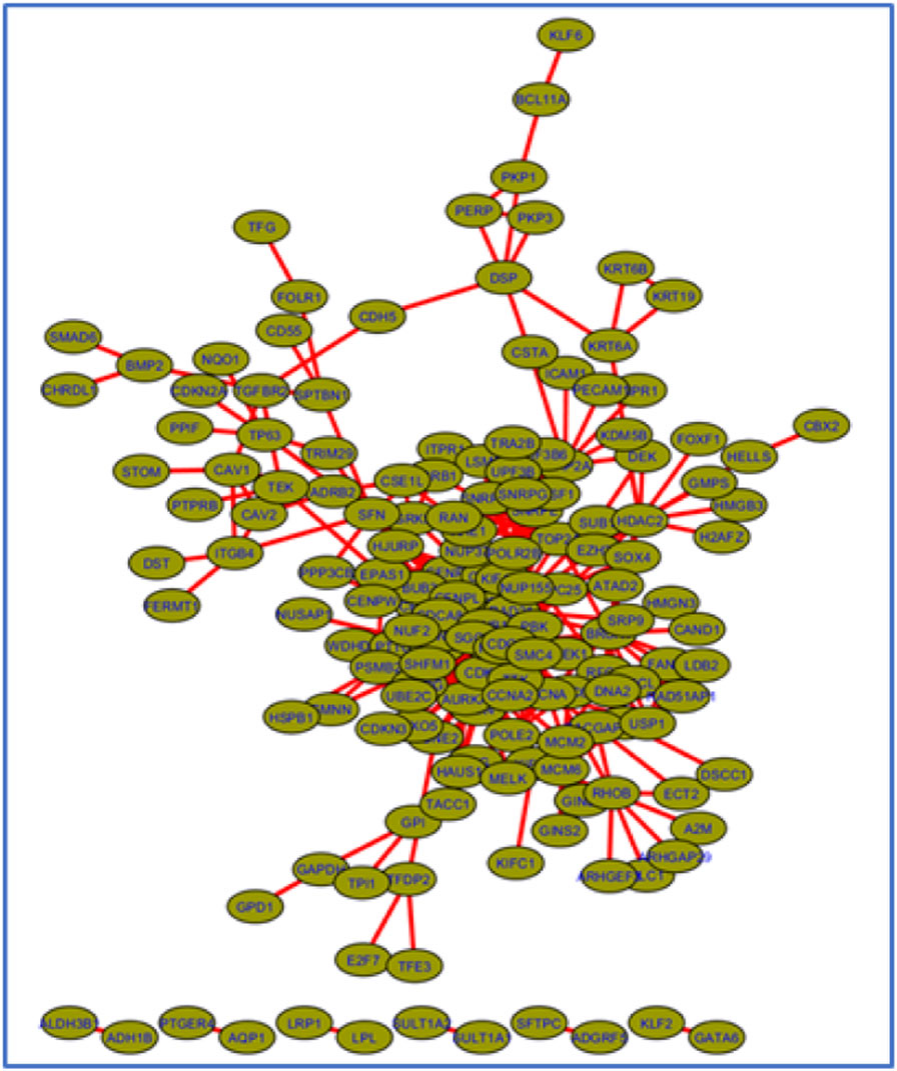
Functionally interacting protein network of non-treatment DEGs created using ReactomeFI

The functionally interacting protein network constructed using non-treatment DEGs has 166 genes and 534 interactions as shown in Fig. 3. Two hundred forty-one out of 407 DEGs for non-treatment studies do not have any functional interaction based on Reactome database. So, they are not included in the network. The reason might be that those genes are not related to any pathways or they are yet to be determined whether they belong to any pathway or not. Further study is required to position those genes on appropriate pathways. Similarly, only 260 out of 547 DEGs are found in Reactome database with 648 functional interaction for treatment studies.

### Biomarker genes

**Fig. 4:**
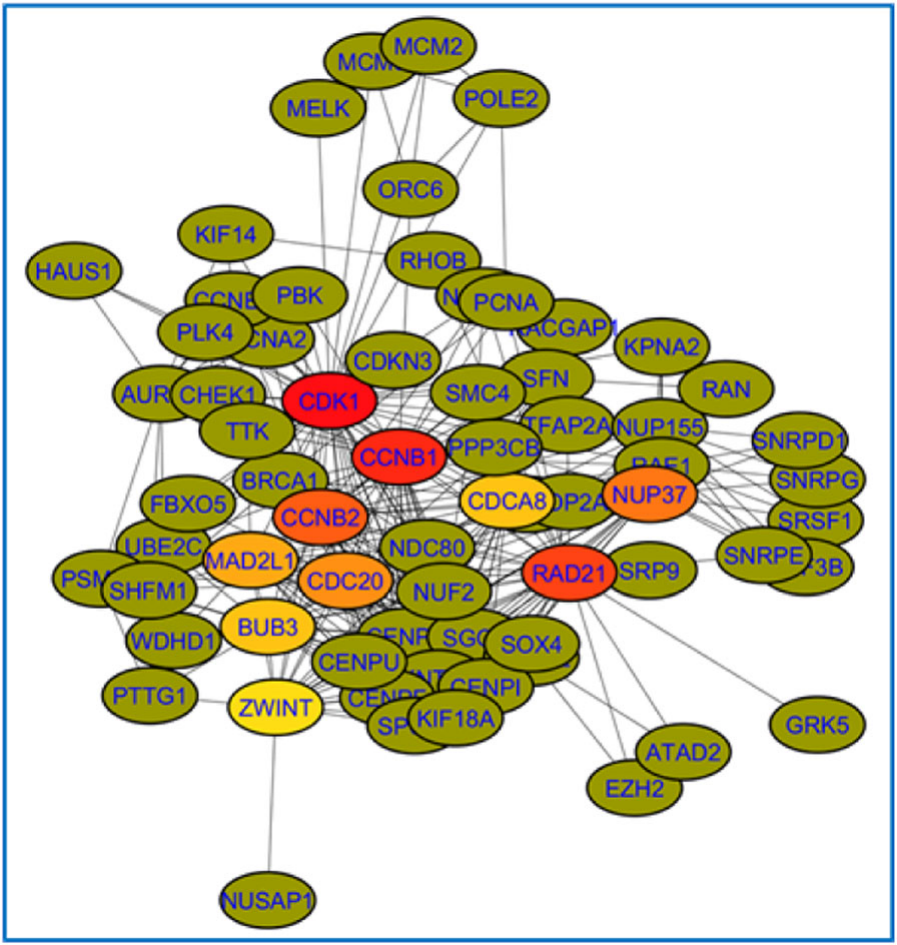
Top 10 hub genes highlighted in non-treatment network. These hub genes are identified using scoring method “Degree” in Cytohubba. The node with dark red represents the highest rank while the node with light yellow represents the lowest rank

Two Cytoscape apps, CytoHubba and MCODE are used to isolate the biomarker genes from the functional networks. Figure 4 shows the top 10 hub genes highlighted in non-treatment network based on the scoring method “Degree” in CytoHubba. The list of hub genes discovered using 12 scoring methods in CytoHubba from non-treatment and treatment studies are shown in Tables 1 *and* 2 *of* Additional File 1.

The app MCODE was used to extract the clusters of genes (probable biomarkers) from the functional networks. A total of 8 and 10 clusters were extracted from non-treatment and treatment networks respectively. The complete list of MCODE clusters with their score, number of nodes and edges is available in Tables 1 *and* 2 *of* Additional File 2.

Any gene common between at least two scoring methods and presents in at least one of the MCODE clusters was considered to be a biomarker gene. A total of 32 biomarker genes - 16 from non-treatment studies and 16 from treatment studies - were discovered in this study.

Tables 2 and 3 show biomarker genes along with their regulation and description in non-treatment and treatment studies respectively. There are no biomarker gene in common between non-treatment and treatment studies.

**Table 2:** The biomarker genes discovered from non-treatment studies along with their regulation and description

Gene	Regulation	Description
BUB3	UP	BUB3 mitotic checkpoint protein
CCNB1	UP	Cyclin B1
CCNB2	DOWN	Cyclin B2
CDC20	DOWN	Cell division cycle 20
CDCA8	DOWN	Cell division cycle associated 8
CDK1	DOWN	Cyclin-dependent kinase 1
CENPF	UP	Centromere Protein F
CENPI	UP	Centromere Protein I
KIF18A	UP	Kinesin Family Member 18A
KNTC1	UP	Kinetochore Associated 1
MAD2L1	UP	MAD2 mitotic arrest deficient like 1
NDC80	UP	NDC80 Kinetochore Complex Component
NUP37	DOWN	Nucleoporing 37 kDa
PCNA	UP	Proliferating Cell Nuclear Antigen
RAD21	UP	RAD21 homolog
ZWINT	UP	ZW10 Interacting Kinetochore Protein

**Table 3:** The biomarker genes discovered from treatment studies along with their regulation and description

Gene	Regulation	Description
CEBPB	DOWN	CCAAT/enhancer binding protein beta
FBXL14	DOWN	F-box and leucine rich repeat protein 14
FBXL3	DOWN	F-box and leucine rich repeat protein 3
FBXO30	DOWN	F-box protein 30
FBXO9	DOWN	F-Box protein 9
FOXA1	DOWN	Forkhead box A1
FOXA2	DOWN	Forkhead box A2
JUN	UP	Jun proto-oncogene AP-1 transcription factor subunit
JUND	DOWN	JunD proto-oncogene, AP-1 transcription factor subunit
MAPK8	UP	Mitogen activated protein kinase 8
MYC	DOWN	V-myc avian myelocytomatosis viral oncogene homolog
MYLIP	DOWN	Myosin regulatory light chain interacting protein
NFE2L2	DOWN	Nuclear factor, erythroid 2 like 2
RNF19A	DOWN	Ring finger protein 19A, RBR E3 ubiquitin protein ligase
RNF217	DOWN	Ring finger protein 217
UBC	DOWN	Ubiquitin C

#### Enriched GO terms and pathways

The biomarker genes of non-treatment and treatment studies were analyzed for enriched pathways and GO terms using GSEApy.

***Non-Treatment Studies:*** The GO terms enriched with biomarker genes from non-treatment studies are shown in Table 1 *of* Additional File 3. The significantly enriched ***biological processes*** are- regulation of mitotic cell cycle phase transition, metaphase plate congression, negative regulation of ubiquitin-protein ligase activity involved in mitotic cell cycle, regulation of ubiquitin-protein ligase activity involved in mitotic cell cycle, positive regulation of ubiquitin-protein ligase activity involved in regulation in mitotic cell cycle, negative regulation of ubiquitin protein ligase activity, anaphase-promoting complex-dependent catabolic process, positive regulation of ubiquitin protein ligase activity, mitotic sister chromatid segregation and positive regulation of protein ubiquitination involved in ubiquitin-dependent protein catabolic process. The significantly enriched ***cellular components*** are- spindle, chromosome, centromeric region, chromosomal region, spindle microtubule, nuclear chromosome part, kinetochore microtubule, centrosome, condensed nuclear chromosome kinetochore, microtubule organizing center and mitotic spindle. The significantly enriched ***molecular functions*** are- cyclin dependent protein serine/threonine kinase activity and cyclin dependent protein kinase activity. The KEGG pathways enriched with biomarker genes from non-treatment studies are shown in Table 1 *of* Additional File 4*.* The significantly enriched ***KEGG pathways*** are- cell cycle, oocyte meiosis, progesterone-mediated oocyte maturation, human T-cell leukemia virus infection, p53 signaling pathway, cellular senescence, human immunodeficiency virus 1 infection, FoxO signaling pathway, viral carcinogenesis and mismatch repair.

**Fig. 5:**
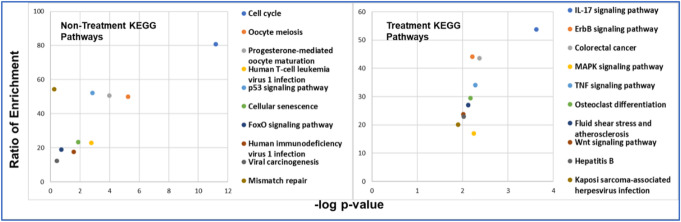
Pathway enrichment analysis of non-treatment and treatment biomarker genes. The significantly enriched KEGG pathways. Each point represents a pathway. Ratio of enrichment is the number of observed genes in that pathway divided by the total number of expected genes from each KEGG pathway

***Treatment Studies:*** The GO terms enriched with biomarker genes from treatment studies are shown in Table 2 *of* Additional File 3. The significantly enriched ***biological process*** are – protein polyubiquitination, protein ubiquitination, positive regulation of transcription from RNA polymerase II promoter, ubiquitin-dependent protein catabolic process, positive regulation of transcription DNA templated, positive regulation of apoptotic process, response to cytokine, regulation of transcription from RNA polymerase II promoter, protein modification by small protein conjugation, and cellular response to reactive oxygen species. The significantly enriched ***cellular components*** are RNA polymerase II transcription factor complex, SCF ubiquitin ligase complex, nuclear chromatin, chromatin, nuclear chromosome part, cullin-RING ubiquitin ligase complex, nuclear euchromatin, euchromatin, nuclear chromosome, and centrosome. The significantly enriched ***molecular functions*** are –ubiquitin-protein transferase activity, transcription regulatory region DNA binding, regulatory region binding, activating transcription factor binding, RNA polymerase II regulatory region sequence-specific DNA binding, DNA binding, RNA polymerase II core promoter proximal region sequence-specific DNA binding, transcription factor activity, and ubiquitin conjugating enzyme binding. The KEGG pathways enriched with biomarker genes from treatment studies are shown in Table 2 *of* Additional File 4*.* The significantly enriched ***KEGG pathways*** are IL-17 signaling pathway, ErbB signaling pathway, colorectal cancer, MAPK signaling pathway, TNF signaling pathway, osteoclast differentiation, fluid shear stress and atherosclerosis, WNT signaling pathway, Hepatitis B and Kaposi sarcoma-associated herpesvirus infection. Figure 5 shows the pathways enriched with biomarker genes from both non-treatment and treatment studies respectively.

### Survival analysis results

**Fig. 6:**
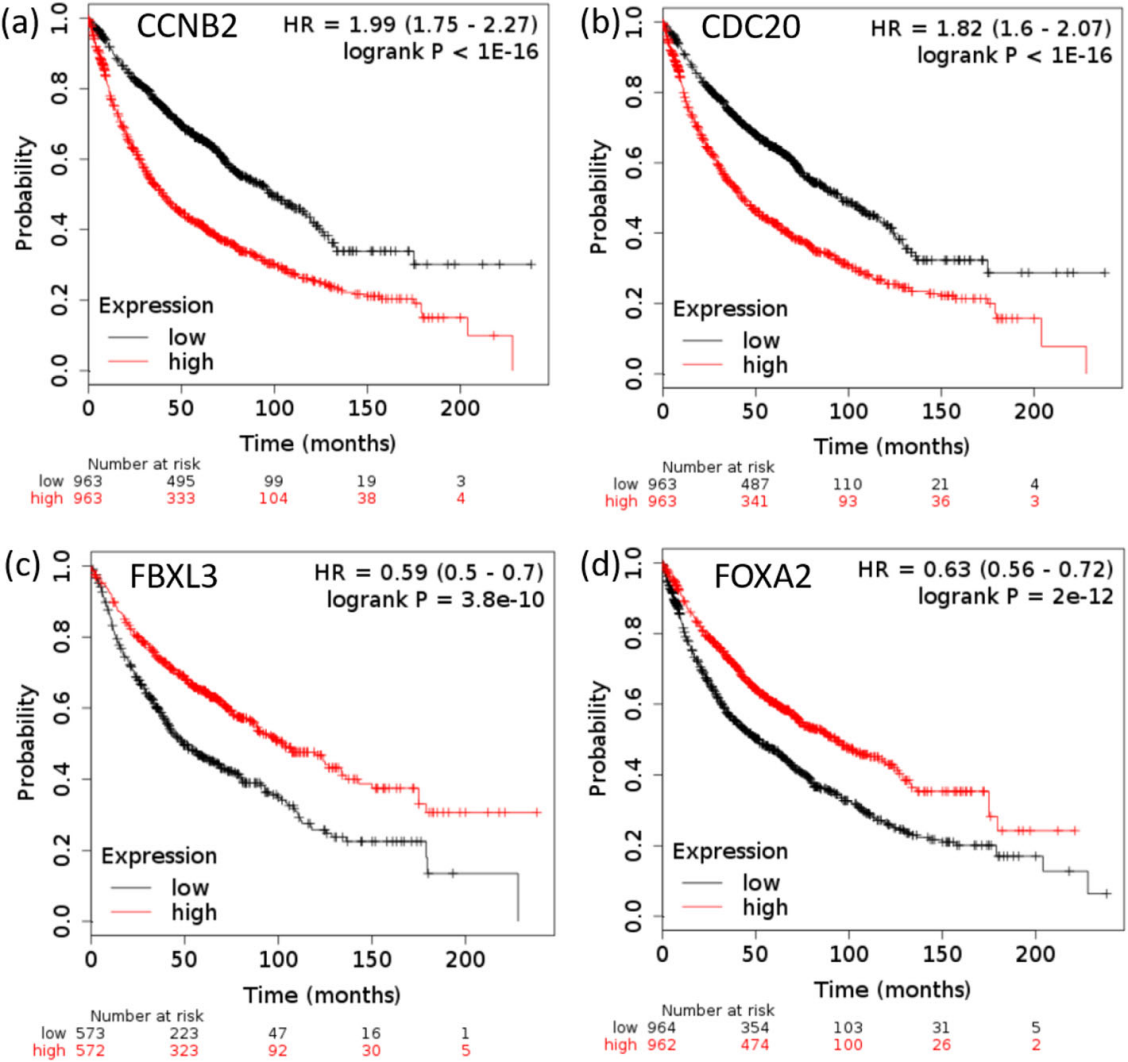
Survival analysis of non-treatment and treatment biomarker genes. a) CCNB2 and b) CDC20 are biomarker genes for non-treatment studies. Both genes have HR > 1, which indicates that low expression group has higher chance of survival. c) FBXL3 and d) FOXA2 are biomarker genes for treatment studies. Both genes have HR < 1, which indicates that high expression group has higher chance of survival

Figure 6 presents the survival analysis results of two non-treament biomarkers (CCNB2 and CDC20) and two treatment biomarkers (FBXL3 and FOXA2). It is clear from this figure that the hazard ratio, HR > 1 for the two non-treament biomakers, which means that low expression group has higher chance of survival compare to high expression group. On the other hand, HR < 1 for two treatment biomarkers, which means that high expression group has higher chance of survival compare to low expression group.

The results of complete survival analysis using each of 16 non-treatment biomarker genes are shown in Fig. 1*of* Additional File 5. Similarly, Fig. 2*of* Additional File 5 shows the survival analysis using 16 treatment biomarker genes. In case of non-treatment studies, 2 biomarkers (CDK1 and PCNA) out of 16, do not have prognostic capability of differentiating between high-expression and low-expression groups of cancer patients, meaning hazard ratio is close to 1 (HR ≈1); 2 biomarkers (BUB3 and RAD21) show that high-expression groups have higher chance of survival (HR <1); and the remaining 12 biomakers show that low-expression groups have higher chance of survival (HR >1).

In case of treatment studies, 2 biomarkers (FOXA1 and JUND) out of 16, do not have prognostic capability of differentiating between high-expression and low-expression groups of cancer patients, meaning hazard ratio is close to 1 (HR ≈1); 3 biomarkers (CEBPB, MYC and UBC) show that low-expression groups have higher chance of survival (HR >1); and the remaining 11 biomakers show that high-expression groups have higher chance of survival (HR <1).

It can be conlcuded that most of the non-treatment biomarker genes (12 out of 16) have prognostic capability by indicating that low-expression groups have higher chance of survival compare to high-expression groups (HR >1). On the other hand, most of the treament biomarker genes (11 out of 16) have prognostic capability by indicating that high-expression groups have higher chance of survival compare to low-expression groups (HR <1). The opposite prognostic characteristics of biomarker genes discovered from non-treatment and treatment studies are expected since in non-treatment studies, controls are healthy samples and cases are cancer patients; whereas, in treatment studies, controls are cancer cell lines without treament and cases are cancer cell lines with treament.

### Discovery of biomarker genes in a nutshell

Table 4 shows the granularity of discovered DEGs at different level of analysis. The GEO built-in filter (up/down genes) retrieved a total of 16,876 DEGs for lung cancer considering all the studies available in GEO database. Based on the five studies (3 non-treatment and 2 treatment) considered in this work, GEO2R tools isolated 407 non-treatment and 547 treatment DEGs. There were 166 non-treatment DEGs and 260 treatment DEGs connected in functional networks based on ReactomeFI database.

**Table 4:** Level of granularity of number of DEGs in different steps of analysis. Each row represents a step of analysis. The second from the last row represents the number of biomarker genes discovered from non-treatment and treatment studies. The last row shows the number of biomarker genes that could be used to design a lab experiment for further exploration of dynamics in lung cancer development

Tools/Analysis	Number of DEGs	
	***Non-Treatment***	***Treatment***
GEO Built-in Filter	16,876	
GEO2R	407	547
ReactomeFI	166	260
Cytohubba	38	51
Hub genes (Common in two algorithms)	21	24
MCODE (Genes present in clusters)	63	55
Biomarkers (Common in Hub and MCODE)	16	16
Survival Analysis	14	14

The 12 scoring methods (algorithms) in Cytohubba were used to find top 10 hub genes from non-treatment and treatment networks. The combined lists of hub genes for non-treatment and treatment studies are 38 and 51 respectively. Hub genes common in at least two scoring methods were selected as probable biomarkers. This resulted in a total of 21 and 24 hub genes for non-treatment and treatment studies respectively. The App, MCODE was used to find clusters in non-treatment and treatment protein networks. There was a total of 8 clusters consisting of 63 genes from non-treatment network and 10 clusters consisting of 55 genes from treatment network. The genes in common between hub genes and MCODE clusters are considered as the biomarker genes. There were 16 biomarker genes from each of non-treatment and treatment studies with no overlap between the two groups. Finally, based on survival analysis, 14 biomarker genes from each of non-treatment and treatment studies have prognostic capability of differentiating between low-expression and high-expression groups of cancer patients. These final lists of biomarkers can be used to design a lab experiment for further exploration of dynamics in lung cancer development.

## Discussion

Of enormous scientific interest has been the discovery of somatic driver mutations in this malignancy, with up to 10 and 4% of non-small cell lung cancers carrying epidermal growth factor receptor mutations and ALK translocations, respectively. The use of tyrosine kinase inhibitors targeting these subpopulations have resulted in significant gains in antitumor responses, progression free survival, overall survival and quality of life shifting patterns of clinical practice [38–41]. More recently, another modality of therapy, inhibition of immune checkpoints such as programmed cell death-1 (PD-1) and programmed cell death ligand-1 (PD-L1) has been also shown to benefit patients with lung cancer. Despite the significant survival benefit for some patients with advanced NSCLC, the objective response rates (ORRs) remain relatively low (20–30%) with a large proportion of patients demonstrating primary resistance [42–44]. However, the low survival rate of lung cancer patients indicates the need for better cancer diagnosis and treatment approaches. The reasons behind low survival of lung cancer patients is the late diagnosis and resistance to chemotherapeutic drugs. There is a need for better therapeutic measures and diagnostic approaches for improving the quality of life and survival of patients. The advancement in biotechnological tools and bioinformatics has changed the course of lung cancer research towards identifying underlying causes of cancer such as molecular pathways and biomarkers. This study used gene expression profiles from GEO datasets to identify DEGs based on two study groups - Non-treatment (healthy samples as control and lung cancer patients as case) and Treatment (lung cancer cell lines without treatment as control and lung cancer cell lines treated with therapeutic drugs as case). Cytoscape apps, Cytohubba and MCODE were used to identify probable biomarker genes from non-treatment and treatment studies which could be diagnostic or therapeutic biomarkers for lung cancer. The following subsections discuss the roles of discovered biomarkers in lung cancer.

### Literatures supporting non-treatment biomarker genes

This section discusses the roles of 16 biomarker genes - BUB3, CCNB1, CCNB2, CDC20, CDCA8, CDK1, CENPF, CENPI, KIF18A, KNTC1, MAD2L1, NDC80, NUP37, PCNA, RAD21, and ZWINT - related to lung cancer discovered from non-treatment studies.

Most of the biomarkers identified in non-treatment studies are associated with biological process mitotic cell cycle (Table 1 *in* Additional File 3) and they are also enriched in pathways such as cell cycle, oocyte meiosis, progesterone-mediated oocyte maturation, and cellular senescence as shown in Table 1 *in* Additional File 4*.* There are hundreds of mutations in our genes every day, some of which might cause deleterious effects. However, most of us are healthy and enjoying our day to day life. This is possible due to delicate checks and balances maintained by the cell regulatory systems. The cell cycle and DNA repair mechanisms are efficient systems which ensure that defective cells are destroyed and only healthy cells remain in our body. The failure in these mechanisms will result in mutations which may lead to genomic abnormalities that lead to cancer. The genes involved in the cell cycle play a vital role in preventing genomic abnormalities. The studies have shown that abnormalities in these genes promote tumorigenesis [45, 46]. An in vivo study in *Drosophila* with knocked down BUB3 showed that the absence of BUB3 induces tumorigenesis [47]. RAD21 is another important protein in a multi-protein complex that plays important role in mitosis [48]. A study by Ni et al., also identified CCNB1, CCNB2, CDK1 and MAD2L1 as key genes for SCLC due to their role during mitosis [49]. A study by Soria et al. showed the overexpression of CCNB1 in both NSCLC and SCC [50]. CCNB1 is a known regulatory protein in mitosis and is necessary for control of G2/M transition phase in cell cycle. CDK1, a cyclin dependent kinase (CDKs), plays an important role in cell progression [51].

CENPF, CENPI, KNTC1, NDC80, and ZWINT are components of kinetochore complex [52]. A study showed the upregulation of CENPF was linked to cancer progression and lymph node metastasis [53]. Another study showed similar link between overexpression of CENPI and colorectal cancer metastasis and progression [54]. The results in this study also showed that the genes CENPF, CENPI, KNTC1, NDC80 and ZWINT are upregulated in non-treatment studies, Table 2.

A study showed that overexpression of PCNA promotes cell proliferation and tumorigenesis in lung cancer [55]. PCNA was found to be up regulated in this study, Table 2.

### Literatures supporting treatment biomarker genes

This section discusses the roles of 16 biomarker genes - CEBPB, FBXL14, FBXL3, FBXO30, FBXO9, FOXA1, FOXA2, JUN, JUND, MAPK8, MYC, MYLIP, NFE2L2, RNF19A, RNF217, and UBC - related to lung cancer discovered from treatment studies.

Most of the biomarkers identified in treatment studies are associated with biological process ubiquitination, Table 2 *in* Additional File 3. Ubiquitination is a post translational modification which is important for maintaining various physiological processes and decides the fate of the protein by marking them for degradation, change in cellular location, promote its activity or preventing protein interaction [56]. UBC (Ubiquitin C) encodes polyubiquitin which plays important role in regulation of cell cycle, DNA repair and kinase activation [57]. UBC is one of the biomarkers discovered from treatment studies. It is down regulated in this study (Table 3) and was found in 10 out of 12 CytoHubba scoring matrices as a top gene (gene with the highest score), Table 2 *in* Additional File 1. MYLIP and RNF19A are both E3 ubiquitin ligase proteins, which are also upregulated, Table 3. F-box protein motifs function as a site of protein-protein interaction [58]. There are four F-box proteins - FBXL14, FBXL3, FBXO30, and FBXO9 - identified as biomarkers in this study, all of which are downregulated, Table 3. F-box proteins are found in cancers as oncogenes or tumor suppressor genes depending upon their expression in their substrate [58].

In lung cancer, FOXA1 and FOXA2 is involved in regulation of Epithelial to Mesenchymal genes relevant to cellular adhesion and cellular communication, and associated with distant metastasis [59–61].

NRF2 (also known as NFE2L2) is a basic leucine zipper transcription factor that regulates the expression of more than 200 genes that protects against stress [62]. In lung cancer NRF2 acts as both oncogene and tumor suppressor gene depending upon the stage of tumor progression [63]. As an oncogene it promotes resistance to chemotherapy and prevents oncogenesis as a tumor suppressor [63]. CEBPB is also a basic leucine zipper transcription factor and it regulates genes that are involved in immune and inflammatory processes [64].

JUN and JUND are signal transducing transcription factors of the AP-1 family and proto oncogenes associated with apoptosis [65]. Levresse et al. [66] and other studies have reported the protective response of c-Jun and JNK pathway in SCLC cells. MAPKs (Mitogen-activated protein kinases) are protein Ser/Thr kinases that convert external stimuli into a wide range of cellular responses [67]. MAPK8 is a protein kinase known as Jun N-terminal kinase-1 (JNK-1) involved in cellular responses to stress [68].

A study by Rapp et.al, showed metastasis in NSCLC driven by MYC gene in a mouse model [69]. Another study by Mollaglu et al. in mouse model showed similar metastasis and tumorigenesis promoted by MYC gene in SCLC [70].

In summary, most of the biomarkers in non-treatment studies are associated with cellular progression and cell cycles (Table 1 *in* Additional File 3) and are upregulated (Table 2). On the other hand, most of the biomarkers in treatment studies are associated with ubiquitination (Table 2 *in* Additional File 3) and are downregulated (Table 3), which affects the activity and progression of proteins. Further study into the molecular mechanisms of these biomarker genes would help to understand the cause of lung cancer as well as the cause of drug resistance in lung cancer. A study in transcriptional landscape of human cancers which studied 33 TCGA cancer types identified that cell cycle pathways and genes related to these pathways are usually upregulated in cancers than in normal tissue [71], which support the findings using non-treatment studies in the present work. Another study by Danielsson et al., also reported up regulation of cell-cycle genes in cancer cells and downregulation of functionally diverse genes in cancer [46], which also support the findings in the present work using both non-treatment and treatment studies.

## Conclusion

This study developed a computational framework to discover biomarker genes for lung cancer using gene expression profiles from GEO database. Two different types of studies – non-treatment and treatment – are considered for experiment. A total of 32 biomarker genes - 16 from non-treatment studies and 16 from treatment studies - were discovered in this study. The results show that most of the non-treatment biomarker genes (12 out of 16) have prognostic capability by indicating that low-expression groups have higher chance of survival compare to high-expression groups. On the other hand, most of the treament biomarker genes (11 out of 16) have prognostic capability by indicating that high-expression groups have higher chance of survival compare to low-expression groups. The opposite prognostic characteristics of biomarker genes discovered from non-treatment and treatment studies are expected since in non-treatment studies, controls are healthy samples and cases are cancer patients; whereas, in treatment studies, controls are cancer cell lines without treament and cases are cancer cell lines with treament. Most of the biomarkers in non-treatment studies (11 out of 16) were upregulated while most of the biomarkers in treatment studies (14 out of 16) were downregulated.

The biomarker genes identified from non-treatment studies play vital role in tumor progression and metastasis. These biomarker genes are associated with cell cycle and consistent with their role in preventing genomic instabilities. The deletion or mutation of these genes induce tumorigenesis. The biomarker genes identified in treatment studies are associated with ubiquitination and response to stress. Ubiquitination is a multistep process for regulation of function and signaling of cellular pathways and cancer cells exploit these pathways for their survival and metastasis [72]. A better understanding of ubiquitination process and its underlying pathways may help to develop better treatment strategies for lung cancer patients.

The scope of this study was limited to computational identification of biomarkers for lung cancer. The existing literatures support that most of the discovered biomarkers play role in tumorigenesis. A detailed study into the roles of these biomarkers and their mechanism of action is required to understand their contribution to tumor progression and survival of lung cancer patients. The biomarker genes discovered in this study could be used for diagnosis and developing appropriate therapeutic approach for the cure of lung cancer. Further biological experiments with the discovered biomarkers are required to validate the findings in this study.

## Supplementary information

**Additional file 1.** Results from CytoHubba. Top 10 hub genes from 12 scoring methods in Cytohubba for non-treatment and treatment studies.

**Additional file 2.** Results from MCODE. The communities obtained from PPI networks of non-treatment and treatment studies.

**Additional file 3.** Enriched GO Terms. The top 10 significant GO terms in 3 categories – Biological Process, Cellular Component, and Molecular Function – enriched with non-treatment and treatment biomarkers.

**Additional file 4.** Enriched Pathways. The top 10 significant pathways enriched with non-treatment and treatment biomarkers.

**Additional file 5.** Survival Analysis Results. The survival analysis results of each individual biomarker gene from both non-treatment and treatment studies.

## Data Availability

The data analyzed in this study was downloaded from NCBI GEO. https://www.ncbi.nlm.nih.gov/geo/

## Abbreviations

caBIG: Cancer Biomedical Informatics Grid
CC: Clustering Coefficient
DEGs: Differentially Expressed Genes
DMNC: Density of Maximum Neighborhood Component
EcC: Eccentricity
EPC: Edge Percolated Component
FC: Fold Change
GE: Gene Expression
GEO: Gene Expression Omnibus
GO: Gene Ontology
GSEA: Gene Set Enrichment Analysis
GSEApy: Gene Set Enrichment Analysis in Python
HR: Hazard Ratio
KEGG: Kyoto Encyclopedia of Gene and Genomes
KM: Kaplan Meier
MCC: Maximal Clique Centrality
MCODE: Molecular Complex Detection
MNC: Maximum Neighborhood Component
MGd: Motexafin Gadolinium
NSCLC: Non-Small Cell Lung Carcinoma
ORR: Objective Response Rates
SCLC: Small Cell Lung Carcinoma
TCGA: The Cancer Genome Atlas

## References

[1] Siegel RL, Miller KD, Jemal A (2019). Cancer statistics, 2019. CA Cancer J Clin.

[2] Larsen JE, Pavey SJ, Passmore LH, Bowman R, Clarke BE, Hayward NK (2007). Expression profiling defines a recurrence signature in lung squamous cell carcinoma. Carcinogenesis..

[3] Guo H, Chen J, Meng F (2016). Identification of novel diagnosis biomarkers for lung adenocarcinoma from the cancer genome atlas. Int J Clin Exp Med.

[4] Siegel RL, Miller KD, Jemal A (2018). Cancer statistics, 2018. CA Cancer J Clin.

[5] Kim B, Hyun JL, Hye YC, Shin Y, Nam S, Seo G (2007). Clinical validity of the lung cancer biomarkers identified by bioinformatics analysis of public expression data. Cancer Res.

[6] Bandyopadhyay S, Mehta M, Kuo D, Sung M-K, Chuang R, Jaehnig EJ (2010). Rewiring of genetic networks in response to DNA damage. Science..

[7] Mondal AM, Hu J. NetLoc: Network based protein localization prediction using protein-protein interaction and co-expression networks. In: 2010 IEEE International Conference on Bioinformatics and Biomedicine (BIBM). IEEE; 2010. 142–148. doi:10.1109/BIBM.2010.5706553.

[8] Mondal AM, Lin J, Hu J (2011). Network based subcellular localization prediction for multi-label proteins. 2011 IEEE international conference on bioinformatics and biomedicine workshops (BIBMW).

[9] Mondal AM, Hu J. Protein Localization by Integrating Multiple Protein Correlation Networks. In: The 2012 International conference on Bioinformatics & Computational Biology. Las Vegas; 2012. 7. https://cse.sc.edu/~jianjunh/paper/BIOCOMP2012.pdf. Accessed 11 Jul 2019.

[10] Lin J-R, Mondal AM, Liu R, Hu J (2012). Minimalist ensemble algorithms for genome-wide protein localization prediction. BMC Bioinformatics.

[11] Mondal AM, Hu J (2013). Scored protein-protein interaction to predict subcellular localizations for yeast using diffusion kernel. Lect Notes Comput Sci.

[12] Mondal A, Hu J (2014). Network based prediction of protein localisation using diffusion kernel. Int J Data Min Bioinform.

[13] Lee H, Tu Z, Deng M, Sun F, Chen T (2006). Diffusion kernel-based logistic regression models for protein function prediction. OMICS..

[14] Qi Y, Suhail Y, Lin Y, Boeke JD, Bader JS (2008). Finding friends and enemies in an enemies-only network: a graph diffusion kernel for predicting novel genetic interactions and co-complex membership from yeast genetic interactions. Genome Res.

[15] Faisal FE, Milenkovic T (2014). Dynamic networks reveal key players in aging. Bioinformatics..

[16] Bett DK, Mondal AM. Diffusion Kernel to Identify Missing PPIs in Protein Network Biomarker. In: 2015 IEEE international conference on bioinformatics and biomedicine (BIBM). IEEE; 2015. p. 1614–9.

[17] Kevin C, Andrews A, Mondal A. Protein Subnetwork Biomarkers for Yeast Using Brute Force Method. In: Proceedings of the International Conference on Bioinformatics & Computational Biology (BIOCOMP). Stylus Publishing; 2013. p. 218–23. https://www.mendeley.com/catalogue/protein-subnetwork-biomarkers-yeast-using-brute-force-method/. Accessed 11 Jul 2019.

[18] Timalsina P, Charles K, Mondal AM. STRING PPI Score to Characterize Protein Subnetwork Biomarkers for Human Diseases and Pathways. In: 2014 IEEE International Conference on Bioinformatics and Bioengineering. IEEE; 2014. p. 251–6.

[19] Mondal AM, Schultz CA, Sheppard M, Carson J, Tanvir RB, Aqila T. Graph Theoretic Concepts as the Building Blocks for Disease Initiation and Progression at Protein Network Level: Identification and Challenges. In: 2018 IEEE international conference on bioinformatics and biomedicine (IEEE BIBM). IEEE; 2018. p. 2713–9. 10.1109/BIBM.2018.8621417.

[20] Tanvir RB, Aqila T, Maharjan M, Mamun AA, Mondal AM. Graph Theoretic and Pearson Correlation-Based Discovery of Network Biomarkers for Cancer. Data. 2019;4:81. 10.3390/data.4020081.

[21] Tanvir RB, Maharjan M, Mondal AM. Community Based Cancer Biomarker Identification from Gene Co-expression Network. In: 10th ACM International Conference on Bioinformatics, Computational Biology and Health Informatics (ACM BCB’19). Association for Computing Machinery (ACM); 2019. p. 545–5.

[22] Tanvir RB, Mondal AM. Cancer Biomarker Discovery from Gene Co-expression Networks Using Community Detection Methods. In: 2019 IEEE International Conference on Bioinformatics & Biomedicine (IEEE BIBM). IEEE; 2019. p. 2097–104.

[23] Chin CH, Chen SH, Wu HH, Ho CW, Ko MT, Lin CY (2014). cytoHubba: Identifying hub objects and sub-networks from complex interactome. BMC Syst Biol.

[24] Bader GD, Hogue CWV (2003). An automated method for finding molecular complexes in large protein interaction networks. BMC Bioinformatics..

[25] Shannon P, Markiel A, Ozier O, Baliga NS, Wang JT, Ramage D (2003). Cytoscape: a software environment for integrated models of biomolecular interaction networks. Genome Res.

[26] Maharjan M, Tanvir RB, Chowdhury K, Mondal AM. Determination of biomarkers for diagnosis of lung Cancer using Cytoscape-based GO and pathway analysis. In: International Conference on Bioinformatics and Computational Biology. Las Vegas; 2019. p. 17–23. https://search.proquest.com/openview/73732e9899442d2bfd96ff526cb3a412/1?pq-origsite=gscholar&cbl=1976360. Accessed 5 Sept 2019.

[27] Barrett T, Wilhite SE, Ledoux P, Evangelista C, Kim IF, Tomashevsky M (2012). NCBI GEO: archive for functional genomics data sets—update. Nucleic Acids Res.

[28] Wu G, Dawson E, Duong A, Haw R, Stein L. ReactomeFIViz: a Cytoscape app for pathway and network-based data analysis. F1000Research. 2014. 10.12688/f1000research.4431.1. 10.12688/f1000research.4431.1 PMC4184317 25309732

[29] Magda D, Lecane P, Miller RA, Lepp C, Miles D, Mesfin M (2005). Motexafin gadolinium disrupts zinc metabolism in human cancer cell lines. Cancer Res.

[30] Wang Z, Lecane PS, Thiemann P, Fan Q, Cortez C, Ma X (2007). Synthesis and biologic properties of hydrophilic sapphyrins, a new class of tumor-selective inhibitors of gene expression. Mol Cancer.

[31] Wachi S, Yoneda K, Wu R (2005). Interactome-transcriptome analysis reveals the high centrality of genes differentially expressed in lung cancer tissues. Bioinformatics..

[32] Sato T, Kaneda A, Tsuji S, Isagawa T, Yamamoto S, Fujita T (2013). PRC2 overexpression and PRC2-target gene repression relating to poorer prognosis in small cell lung cancer. Sci Rep.

[33] Pacheco-Pinedo EC, Durham AC, Stewart KM, Goss AM, Lu MM, Demayo FJ (2011). Wnt/beta-catenin signaling accelerates mouse lung tumorigenesis by imposing an embryonic distal progenitor phenotype on lung epithelium. J Clin Invest.

[34] Wu G, Feng X, Stein L (2010). A human functional protein interaction network and its application to cancer data analysis. Genome Biol.

[35] Kuleshov MV, Jones MR, Rouillard AD, Fernandez NF, Duan Q, Wang Z (2016). Enrichr: a comprehensive gene set enrichment analysis web server 2016 update. Nucleic Acids Res.

[36] Subramanian A, Tamayo P, Mootha VK, Mukherjee S, Ebert BL, Gillette MA (2005). Gene set enrichment analysis: a knowledge-based approach for interpreting genome-wide expression profiles. Proc Natl Acad Sci.

[37] Gyorffy B, Surowiak P, Budczies J, Lanczky A (2013). Online survival analysis software to assess the prognostic value of biomarkers using transcriptomic data in non-small-cell lung cancer. PLoS One.

[38] Kwak EL, Bang YJ, Camidge DR, Shaw AT, Solomon B, Maki RG (2010). Anaplastic lymphoma kinase inhibition in non-small-cell lung cancer. [Erratum appears in N Engl J Med. 2011 Feb 10;364(6):588]. N Engl J Med.

[39] Hirsch FR, Scagliotti GV, Mulshine JL, Kwon R, Curran WJ, Wu YL (2017). Lung cancer: current therapies and new targeted treatments. Lancet.

[40] Mok TS, Wu Y-L, Thongprasert S, Yang C-H, Chu D-T, Saijo N (2009). Gefitinib or carboplatin–paclitaxel in pulmonary adenocarcinoma. N Engl J Med.

[41] Kris MG, Johnson BE, Berry LD, Kwiatkowski DJ, Iafrate AJ, Wistuba II (2014). Using multiplexed assays of oncogenic drivers in lung cancers to select targeted drugs. JAMA.

[42] Berland L, Heeke S, Humbert O, Macocco A, Long-Mira E, Lassalle S (2019). Current views on tumor mutational burden in patients with nonsmall cell lung cancer treated by immune checkpoint inhibitors. J Thoracic Dis.

[43] Heeke S, Hofman P (2018). Tumor mutational burden assessment as a predictive biomarker for immunotherapy in lung cancer patients: getting ready for prime-time or not?. Transl Lung Cancer Res.

[44] Cho JH (2017). Immunotherapy for non-small-cell lung cancer: current status and future obstacles. Immune Netw.

[45] Dienstmann R, Jang IS, Bot B, Friend S, Guinney J (2015). Database of genomic biomarkers for cancer drugs and clinical targetability in solid tumors. Cancer Discov.

[46] Danielsson F, Skogs M, Huss M, Rexhepaj E, O’Hurley G, Klevebring D (2013). Majority of differentially expressed genes are down-regulated during malignant transformation in a four-stage model. Proc Natl Acad Sci U S A.

[47] Morais da Silva S, Moutinho-Santos T, Sunkel CE (2013). A tumor suppressor role of the Bub3 spindle checkpoint protein after apoptosis inhibition. J Cell Biol.

[48] Deb S, Xu H, Tuynman J, George J, Yan Y, Li J (2014). RAD21 cohesin overexpression is a prognostic and predictive marker exacerbating poor prognosis in KRAS mutant colorectal carcinomas. Br J Cancer.

[49] Ni Z, Wang X, Zhang T, Li L, Li J (2018). Comprehensive analysis of differential expression profiles reveals potential biomarkers associated with the cell cycle and regulated by p53 in human small cell lung cancer. Exp Ther Med.

[50] Soria JC, Jang SJ, Khuri FR, Hassan K, Liu D, Hong WK (2000). Overexpression of cyclin B1 in early-stage non-small cell lung cancer and its clinical implication. Cancer Res.

[51] Barnum KJ, O’Connell MJ (2014). Cell cycle regulation by checkpoints. Methods Mol Biol.

[52] Rebhan M, Chalifa-Caspi V, Prilusky J, Lancet D (1998). GeneCards: a novel functional genomics compendium with automated data mining and query reformulation support. Bioinformatics..

[53] Göbel C, Özden C, Schroeder C, Hube-Magg C, Kluth M, Möller-Koop C (2018). Upregulation of centromere protein F is linked to aggressive prostate cancers. Cancer Manag Res.

[54] Ding N, Li R, Shi W, He C (2018). CENPI is overexpressed in colorectal cancer and regulates cell migration and invasion. Gene..

[55] Wang L, Kong W, Liu B, Zhang X (2018). Proliferating cell nuclear antigen promotes cell proliferation and tumorigenesis by up-regulating STAT3 in non-small cell lung cancer. Biomed Pharmacother.

[56] Akimov V, Barrio-Hernandez I, Hansen SVF, Hallenborg P, Pedersen A-K, Bekker-Jensen DB (2018). UbiSite approach for comprehensive mapping of lysine and N-terminal ubiquitination sites. Nat Struct Mol Biol.

[57] Kim M-N, Choi J, Ryu H-W, Ryu K-Y (1853). Disruption of polyubiquitin gene Ubc leads to attenuated resistance against arsenite-induced toxicity in mouse embryonic fibroblasts. Biochim Biophys Acta Mol Cell Res.

[58] Kipreos ET, Pagano M (2000). The F-box protein family. Genome Biol.

[59] Wang H, Meyer CA, Fei T, Wang G, Zhang F, Liu XS (2013). A systematic approach identifies FOXA1 as a key factor in the loss of epithelial traits during the epithelial-to-mesenchymal transition in lung cancer. BMC Genomics.

[60] Song Y, Washington MK, Crawford HC (2010). Loss of FOXA1/2 Is Essential for the Epithelial-to-Mesenchymal Transition in Pancreatic Cancer. Cancer Res.

[61] Tang Y, Shu G, Yuan X, Jing N, Song J (2011). FOXA2 functions as a suppressor of tumor metastasis by inhibition of epithelial-to-mesenchymal transition in human lung cancers. Cell Res.

[62] Huppke P, Weissbach S, Church JA, Schnur R, Krusen M, Dreha-Kulaczewski S (2017). Activating de novo mutations in NFE2L2 encoding NRF2 cause a multisystem disorder. Nat Commun.

[63] Tong Y-H, Zhang B, Fan Y, Lin N-M (2015). Keap1–Nrf2 pathway: a promising target towards lung cancer prevention and therapeutics. Chronic Dis Transl Med.

[64] O’Leary NA, Wright MW, Brister JR, Ciufo S, Haddad D, McVeigh R (2016). Reference sequence (RefSeq) database at NCBI: current status, taxonomic expansion, and functional annotation. Nucleic Acids Res.

[65] Zhang S, Liu Y, Wang Z, Liu J, Gu Z, Xu Q (2017). PAR1-mediated c-Jun activation promotes heat stress-induced early stage apoptosis of human umbilical vein endothelial cells. Mol Med Rep.

[66] Levresse V, Marek L, Blumberg D, Heasley LE (2002). Regulation of platinum-compound cytotoxicity by the c-Jun N-terminal kinase and c-Jun signaling pathway in small-cell lung cancer cells. Mol Pharmacol.

[67] Cargnello M, Roux PP (2011). Activation and function of the MAPKs and their substrates, the MAPK-activated protein kinases. Microbiol Mol Biol Rev.

[68] Chen Y-R, Wang X, Templeton D, Davis RJ, Tan T-H (1996). The role of c-Jun N-terminal kinase (JNK) in apoptosis induced by ultraviolet C and γ radiation duration of JNK activation may determine cell death and proliferation. J Biol Chem.

[69] Rapp UR, Korn C, Ceteci F, Karreman C, Luetkenhaus K, Serafin V (2009). Myc is a metastasis gene for non-small-cell lung Cancer. PLoS One.

[70] Mollaoglu G, Guthrie MR, Böhm S, Brägelmann J, Can I, Ballieu PM (2017). MYC drives progression of small cell lung Cancer to a variant neuroendocrine subtype with vulnerability to Aurora kinase inhibition. Cancer Cell.

[71] Li M, Sun Q, Wang X (2017). Transcriptional landscape of human cancers. Oncotarget..

[72] Gallo LH, Ko J, Donoghue DJ (2017). The importance of regulatory ubiquitination in cancer and metastasis. Cell Cycle.

