# Supplementary material for: Computational identification of biomarker genes for lung cancer considering treatment and non-treatment studies
Source: BMC Bioinformatics. 2020 Dec 3;21(Suppl 9):218. doi: 10.1186/s12859-020-3524-8 (PMC7713218; doi:10.1186/s12859-020-3524-8)
Supplement: Supplementary file 2 — Additional file 2. Results from MCODE. The communities obtained from PPI networks of non-treatment and treatment studies. [file 12859_2020_3524_MOESM2_ESM.pdf]

Additional File 2 – MCODE Clusters

Table 1: MCODE clusters for non-treatment studies. A cytoscape app, MCODE was used to find communities from the non-treatment PPI network. The communities were obtained using a default parameter in MCODE (threshold degree cutoff of 2, node score cutoff:0.2, K-core :2 and max. depth from seed:100)

Cluster	Score	Nodes	Edges	Node IDs
1	20	20	190	CENPL, ZWINT, CDC20, CDK1, RAD21, CCNB1, CENPI, NUF2, SGO2, MAD2L1, CDCA8, NDC80, CCNB2, NUP37, BUB3, CENPF, SPC25, KNTC1, CENPU, KIF18A
2	9	11	45	LSM8, UPF3B, SNRPG, SRSF1, SF3B6, RAE1, SNRPE, POLR2B, NUP155, TRA2B, SNRPD1
3	4.25	9	17	PLK4, CHEK1, CCNA2, PSMB2, TTK, AURKA, PBK, SHFM1, UBE2C
4	4	5	8	POLE2, MCM6, MCM2, ORC6, BRIP1
5	4	5	8	PCNA, USP1, FANCI, FANCL, RFC4
6	3	7	9	KRT19, PERP, PKP3, KRT6A, KRT6B, PKP1, DSP
7	3	3	3	RACGAP1, ECT2, RHOB
8	3	3	3	CAV1, CAV2, ITGB4

Table 2: MCODE clusters for treatment studies. A cytoscape app, MCODE was used to find communities from the treatment PPI network. The communities were obtained using default parameters in MCODE (threshold degree cutoff of 2, node score cutoff:0.2, K-core :2 and max. depth from seed:100)

Cluster	Score	Nodes	Edges	Node IDs
1	10	10	45	FBXO30, UBC, MYLIP, SIAH1, SIAH2, FBXL14, FBXL3, RNF19A, FBXO9, RNF217
2	9	9	36	SRSF7, SRSF3, HNRNPA2B1, PCBP2, SRSF4, CDC40, SRSF1, SNRPA1, HNRNPA1
3	6	6	15	MT2A, MT1X, MT1H, MT1E, MT1G, MT1F
4	5	5	10	IMP3, EXOSC7, NOL11, DIEXF, UTP3

5	4.857	8	17	CBFA2T2, FOXA2, FOXA1, DUSP1, JUN, MAPK8, CEBPB, NFE2L2
6	4	4	6	ERCC4, ERCC2, ZNF830, CDK7
7	4	4	6	PNRC2, SMG8, RPL37A, RPS24
8	3	3	3	KDM5B, MYC, ZNF217
9	3	3	3	WASL, TRIO, NCK1
10	3	3	3	TGFBR2, YAP1, JUND
