# Supplementary material for: Computational identification of biomarker genes for lung cancer considering treatment and non-treatment studies
Source: BMC Bioinformatics. 2020 Dec 3;21(Suppl 9):218. doi: 10.1186/s12859-020-3524-8 (PMC7713218; doi:10.1186/s12859-020-3524-8)
Supplement: Supplementary file 3 — Additional file 3. Enriched GO Terms. The top 10 significant GO terms in 3 categories – Biological Process, Cellular Component, and Molecular Function – enriched with non-treatment and treatment biomarkers. [file 12859_2020_3524_MOESM3_ESM.pdf]

Additional File 3 – Enriched GO Terms

Table 1: GO terms enriched with non-treatment biomarker genes

**Biological Process**	
**Term**	**Genes**
Regulation of mitotic cell cycle phase transition (GO:1901990)	CDC20, CENPF, CCNB1, CDK1, KNTC1, BUB3, MAD2L1
Metaphase plate congression (GO:0051310)	KIF18A, CENPF, CCNB1, CDCA8, NDC80
Negative regulation of ubiquitin-protein ligase activity involved in mitotic cell cycle (GO:0051436)	CDC20, CCNB1, CDK1, BUB3, MAD2L1
Regulation of ubiquitin-protein ligase activity involved in mitotic cell cycle (GO:0051439)	CDC20, CCNB1, CDK1, BUB3, MAD2L1
Positive regulation of ubiquitin-protein ligase activity involved in regulation of mitotic cell cycle transition (GO:0051437)	CDC20, CCNB1, CDK1, BUB3, MAD2L1
Negative regulation of ubiquitin protein ligase activity (GO:1904667)	CDC20, CCNB1, CDK1, BUB3, MAD2L1
Anaphase-promoting complex-dependent catabolic process (GO:0031145)	CDC20, CCNB1, CDK1, BUB3, MAD2L1
Positive regulation of ubiquitin protein ligase activity (GO:1904668)	CDC20, CCNB1, CDK1, BUB3, MAD2L1
Mitotic sister chromatid segregation (GO:0000070)	KIF18A, CCNB1, CDCA8, ZWINT, NDC80
Positive regulation of protein ubiquitination involved in ubiquitin-dependent protein catabolic process (GO:2000060)	CDC20, CCNB1, CDK1, BUB3, MAD2L1
**Cellular Process**	
**Term**	**Genes**
Spindle (GO:0005819)	CDC20, CENPF, CCNB1, CDK1, CDCA8, KNTC1, MAD2L1
Chromosome, centromeric region (GO:0000775)	CENPF, RAD21, CDCA8, NDC80
Chromosomal region (GO:0098687)	CENPF, RAD21, CDCA8, NDC80

Spindle microtubule (GO:0005876)	KIF18A, CDK1, KNTC1
Nuclear chromosome part (GO:0044454)	CCNB1, PCNA, RAD21, CDK1, NDC80
Kinetochore microtubule (GO:0005828)	KIF18A, KNTC1
Centrosome (GO:0005813)	CCNB2, CCNB1, PCNA, CDK1, NDC80
Condensed nuclear chromosome kinetochore (GO:0000778)	CCNB1, NDC80
Microtubule organizing center (GO:0005815)	CCNB2, CCNB1, PCNA, CDK1, NDC80
Mitotic spindle (GO:0072686)	KIF18A, CDK1, MAD2L1
**Molecular Function**	
**Term**	**Genes**
Cyclin-dependent protein serine/threonine kinase activity (GO:0004693)	CCNB2, CCNB1, CDK1
Cyclin-dependent protein kinase activity (GO:0097472)	CCNB2, CCNB1, CDK1

Table 2: GO terms enriched with treatment biomarker genes

**Biological Process**	
**Term**	**Genes**
Protein polyubiquitination (GO:0000209)	MYLIP, RNF19A, UBC, RNF217, FBXL3, FBXL14, FBXO30, FBXO9
Protein ubiquitination (GO:0016567)	MYLIP, RNF19A, UBC, RNF217, FBXL3, FBXL14, FBXO30, FBXO9, NFE2L2
Positive regulation of transcription from RNA polymerase II promoter (GO:0045944)	FOXA1, CEBPB, JUN, JUND, MYC, UBC, FOXA2, NFE2L2
Ubiquitin-dependent protein catabolic process (GO:0006511)	MYLIP, RNF19A, UBC, RNF217, FBXL14, NFE2L2

Positive regulation of transcription, DNA-templated (GO:0045893)	FOXA1, CEBPB, JUN, JUND, MYC, UBC, FOXA2, NFE2L2
Positive regulation of apoptotic process (GO:0043065)	FOXA1, JUN, MAPK8, MYC, UBC
Response to cytokine (GO:0034097)	JUN, MAPK8, JUND, FOXA2
Regulation of transcription from RNA polymerase II promoter (GO:0006357)	FOXA1, CEBPB, JUN, JUND, MYC, UBC, FOXA2, NFE2L2
Protein modification by small protein conjugation (GO:0032446)	MYLIP, UBC, FBXL3, FBXO9, NFE2L2
Cellular response to reactive oxygen species (GO:0034614)	JUN, MAPK8, NFE2L2
**Cellular Component**	
**Term**	**Genes**
RNA polymerase II transcription factor complex (GO:0090575)	CEBPB, JUN, JUND
SCF ubiquitin ligase complex (GO:0019005)	FBXL3, FBXO9
Nuclear chromatin (GO:0000790)	JUN, JUND, MYC
Chromatin (GO:0000785)	JUN, JUND, MYC
Nuclear chromosome part (GO:0044454)	JUN, JUND, MYC
Cullin-RING ubiquitin ligase complex (GO:0031461)	FBXL3, FBXO9
Nuclear euchromatin (GO:0005719)	JUN
Euchromatin (GO:0000791)	JUN
Nuclear chromosome (GO:0000228)	JUN
Centrosome (GO:0005813)	RNF19A, NFE2L2

Molecular Function	
**Term**	**Genes**
Ubiquitin-protein transferase activity (GO:0004842)	MYLIP, RNF19A, RNF217, FBXL3, FBXL14, FBXO30, FBXO9
Transcription regulatory region DNA binding (GO:0044212)	FOXA1, JUN, JUND, MYC, FOXA2, NFE2L2
Regulatory region DNA binding (GO:0000975)	FOXA1, JUN, JUND, FOXA2, NFE2L2
Activating transcription factor binding (GO:0033613)	JUN, MYC, NFE2L2
RNA polymerase II regulatory region sequence-specific DNA binding (GO:0000977)	CEBPB, JUN, JUND, MYC, FOXA2
DNA binding (GO:0003677)	FOXA1, CEBPB, JUN, MYC, FOXA2, NFE2L2
RNA polymerase II core promoter proximal region sequence-specific DNA binding (GO:0000978)	JUN, JUND, MYC, FOXA2
Core promoter proximal region sequence-specific DNA binding (GO:0000987)	JUN, JUND, MYC, FOXA2
Transcription factor activity	FOXA1, CEBPB, JUN, MYC
Ubiquitin conjugating enzyme binding (GO:0031624)	RNF19A, RNF217
