# Supplementary material for: Computational identification of biomarker genes for lung cancer considering treatment and non-treatment studies
Source: BMC Bioinformatics. 2020 Dec 3;21(Suppl 9):218. doi: 10.1186/s12859-020-3524-8 (PMC7713218; doi:10.1186/s12859-020-3524-8)
Supplement: Supplementary file 4 — Additional file 4. Enriched Pathways. The top 10 significant pathways enriched with non-treatment and treatment biomarkers. [file 12859_2020_3524_MOESM4_ESM.pdf]

Additional File 4 - Enriched Pathways

Table 1: KEGG pathways enriched with non-treatment biomarker genes

Term	Genes
Cell cycle	CDC20, CCNB2, CCNB1, PCNA, RAD21, CDK1, BUB3, MAD2L1
Oocyte meiosis	CDC20, CCNB2, CCNB1, CDK1, MAD2L1
Progesterone-mediated oocyte maturation	CCNB2, CCNB1, CDK1, MAD2L1
Human T-cell leukemia virus 1 infection	CDC20, CCNB2, BUB3, MAD2L1
P53 signaling pathway	CCNB2, CCNB1, CDK1
Cellular senescence	CCNB2, CCNB1, CDK1
Human immunodeficiency virus 1 infection	CCNB2, CCNB1, CDK1
FoxO signaling pathway	CCNB2, CCNB1
Viral carcinogenesis	CDC20, CDK1
Mismatch repair	PCNA

Table 2: KEGG pathways enriched with treatment biomarker genes

Term	Genes
IL-17 signaling pathway	CEBPB, JUN, MAPK8, JUND
ErbB signaling pathway	JUN, MAPK8, MYC
Colorectal cancer	JUN, MAPK8, MYC
MAPK signaling pathway	JUN, MAPK8, JUND, MYC
TNF signaling pathway	CEBPB, JUN, MAPK8
Osteoclast differentiation	JUN, MAPK8, JUND
Fluid shear stress and atherosclerosis	JUN, MAPK8, NFE2L2
Wnt signaling pathway	JUN, MAPK8, MYC
Hepatitis B	JUN, MAPK8, MYC
Kaposi sarcoma-associated herpesvirus infection	JUN, MAPK8, MYC
