# Supplementary material for: Computational identification of biomarker genes for lung cancer considering treatment and non-treatment studies
Source: BMC Bioinformatics. 2020 Dec 3;21(Suppl 9):218. doi: 10.1186/s12859-020-3524-8 (PMC7713218; doi:10.1186/s12859-020-3524-8)
Supplement: Supplementary file 5 — Additional file 5. Survival Analysis Results. The survival analysis results of each individual biomarker gene from both non-treatment and treatment studies. [file 12859_2020_3524_MOESM5_ESM.pdf]

Additional File 5 – Survial Analysis

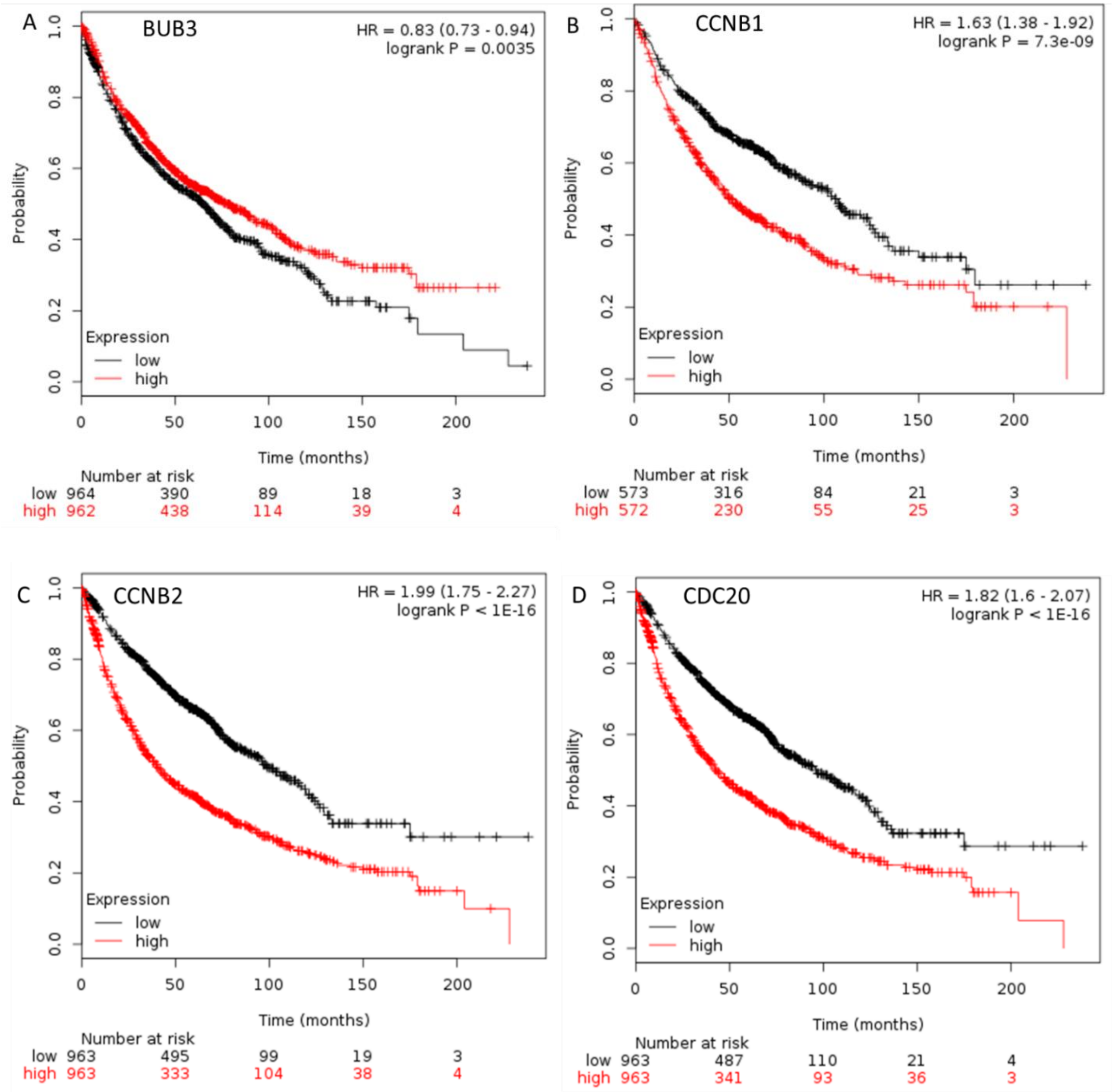

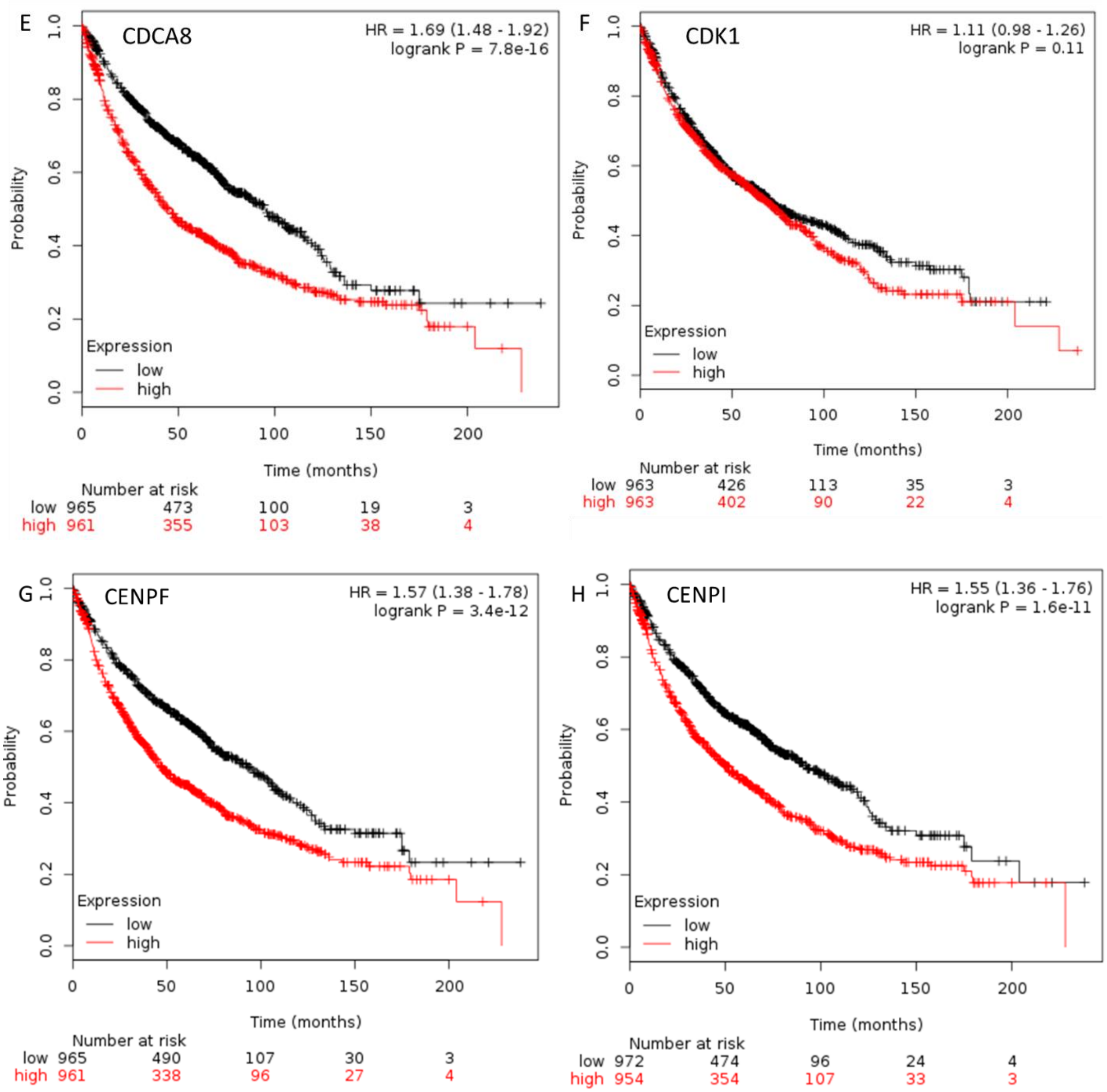
E
CDCA8
HR = 1.69 (1.48 - 1.92)
logrank P = 7.8e-16
Probability
Time (months)
Expression
low
high
Number at risk
low 965 473 100 19 3
high 961 355 103 38 4
F
CDK1
HR = 1.11 (0.98 - 1.26)
logrank P = 0.11
Probability
Time (months)
Expression
low
high
Number at risk
low 963 426 113 35 3
high 963 402 90 22 4
G
CENPF
HR = 1.57 (1.38 - 1.78)
logrank P = 3.4e-12
Probability
Time (months)
Expression
low
high
Number at risk
low 965 490 107 30 3
high 961 338 96 27 4
H
CENPI
HR = 1.55 (1.36 - 1.76)
logrank P = 1.6e-11
Probability
Time (months)
Expression
low
high
Number at risk
low 972 474 96 24 4
high 954 354 107 33 3

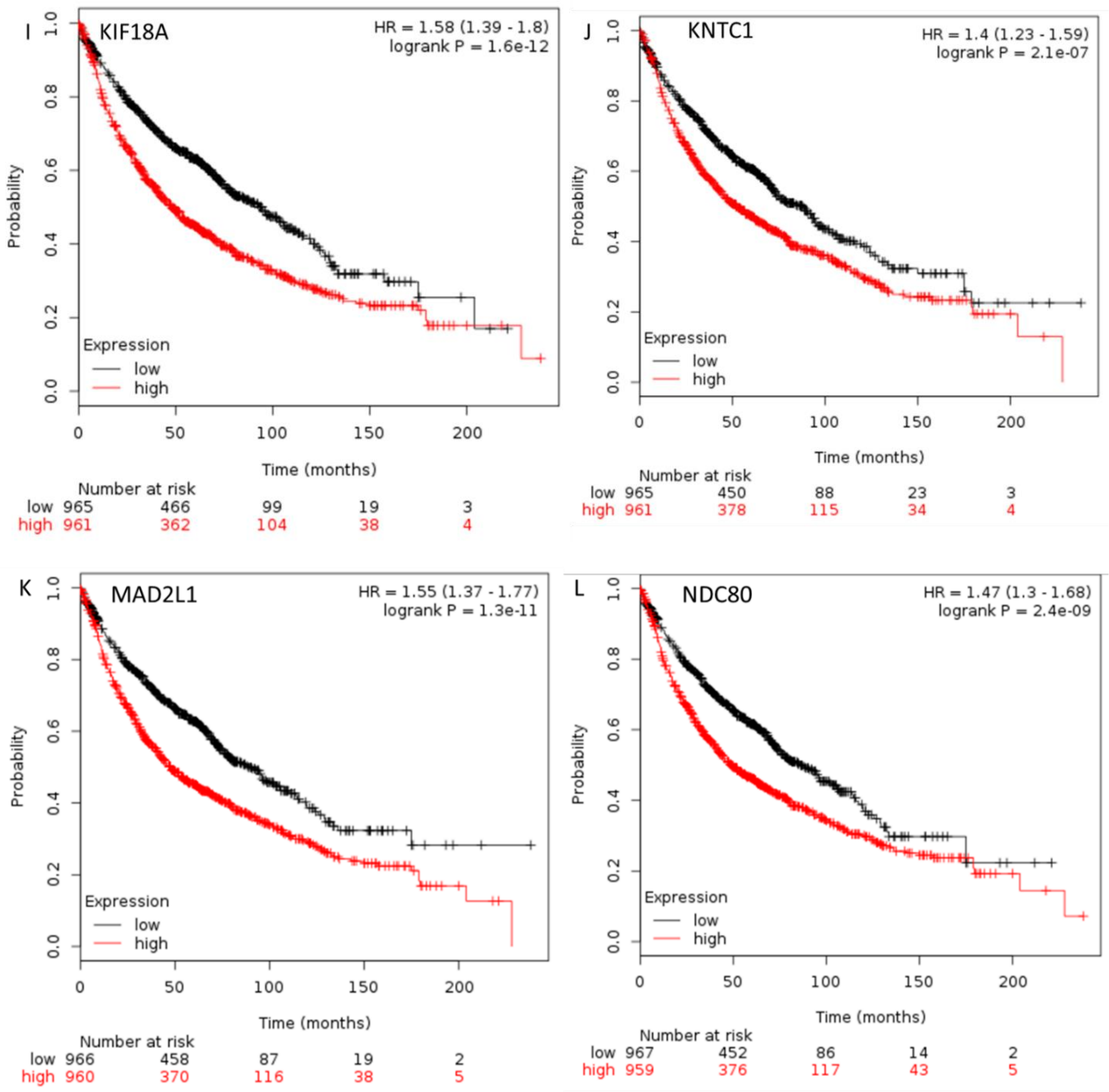
I
KIF18A
HR = 1.58 (1.39 - 1.8)
logrank P = 1.6e-12
Probability
Expression
low
high
Time (months)
Number at risk
low 965 466 99 19 3
high 961 362 104 38 4
J
KNTC1
HR = 1.4 (1.23 - 1.59)
logrank P = 2.1e-07
Probability
Expression
low
high
Time (months)
Number at risk
low 965 450 88 23 3
high 961 378 115 34 4
K
MAD2L1
HR = 1.55 (1.37 - 1.77)
logrank P = 1.3e-11
Probability
Expression
low
high
Time (months)
Number at risk
low 966 458 87 19 2
high 960 370 116 38 5
L
NDC80
HR = 1.47 (1.3 - 1.68)
logrank P = 2.4e-09
Probability
Expression
low
high
Time (months)
Number at risk
low 967 452 86 14 2
high 959 376 117 43 5

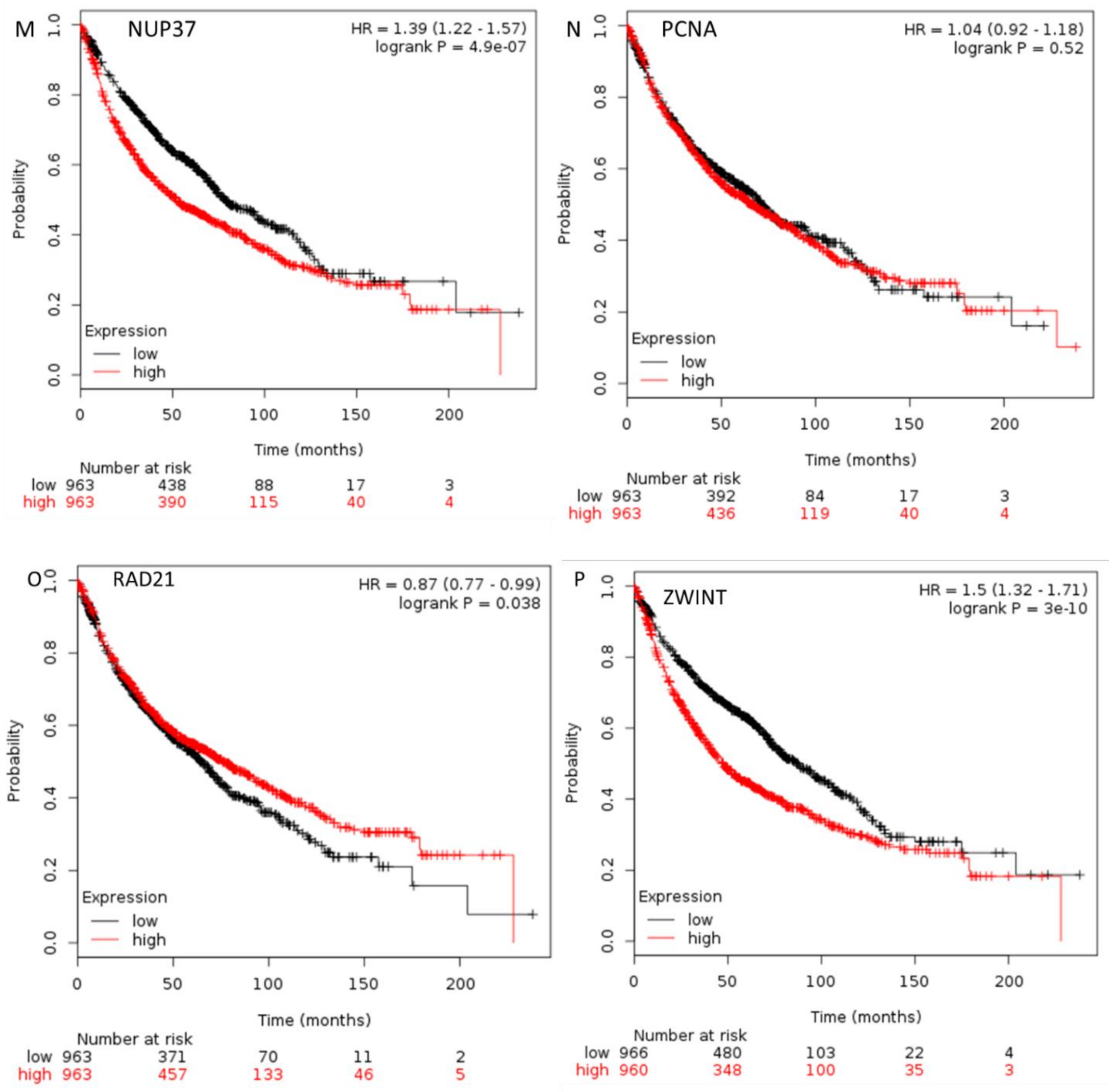

Figure 1: **Survival analysis using non-treatment biomarker genes.** A) BUB3, B) CCNB1, C) CCNB2, D) CDC20, E) CDCA8, F) CDK1, G) CENPF H) CENPI, I) KIF18A, J) KNTC1, K) MAD2L1, L) NDC80, M) NUP37, N) PCNA, O) RAD21 and P) ZWINT. The red and black curve denote samples having gene expression value above or below threshold, which is median for each gene. The hazard ratio (HR) is computed as high-expression group vs low-expression group. X-axis is the timeline in months and Y-axis is the probability of survival of each subgroup. Also, below the X-axis, the number of samples for each subgroup are given at each time point. ***Conclusion:*** 2 biomarkers (CDK1 and PCNA) out of 16, do not have prognostic capability of differentiating between high-expression and low-expression groups of cancer patients, meaning hazard ratio is close to 1 (HR ≈ 1); 2 biomarkers (BUB3 and RAD21) show that high-expression groups have higher chance of survival (HR < 1); and the remaining 12 biomakers show that low-expression groups have higher chance of survival (HR > 1).

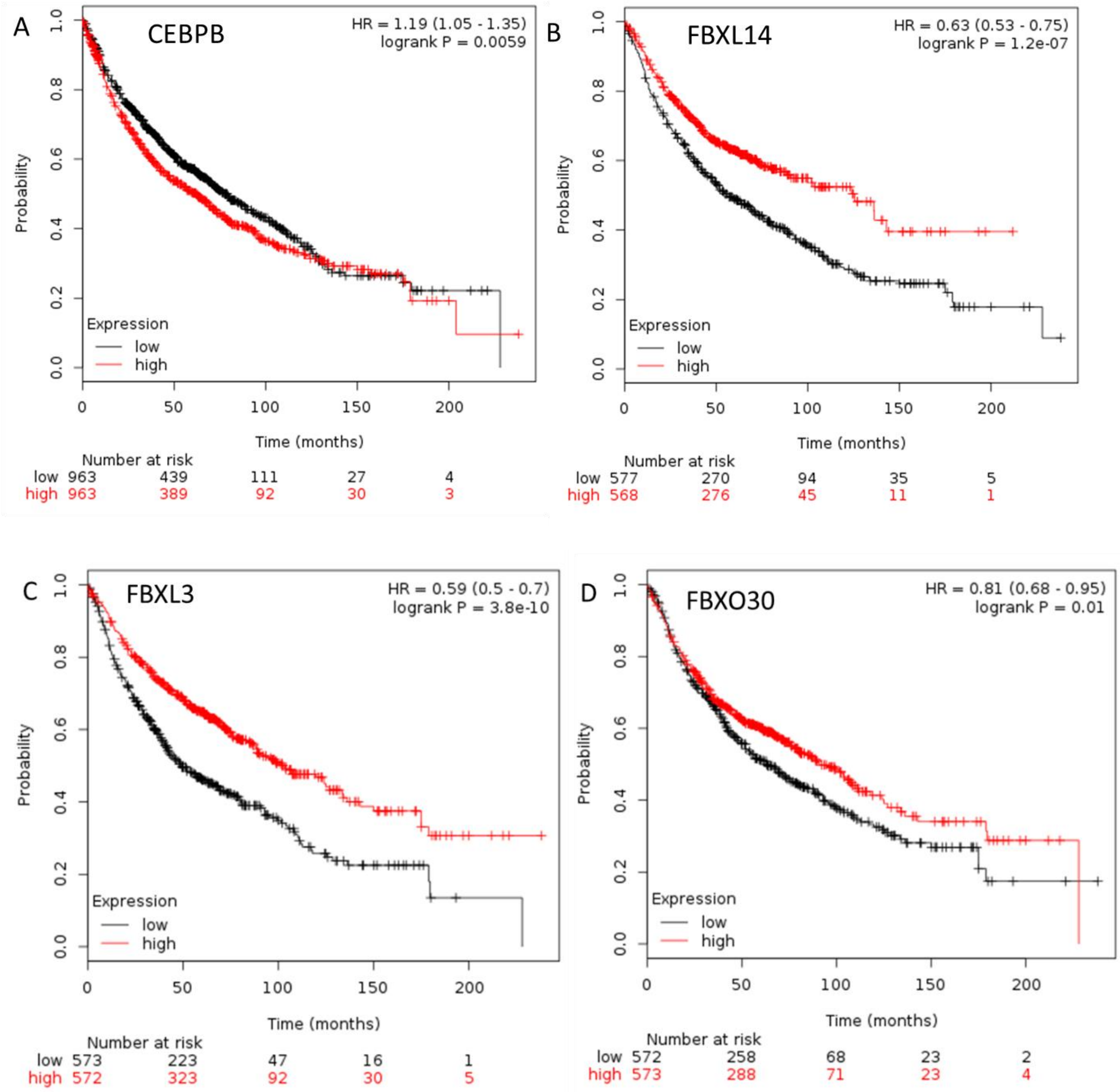
A
CEBPB
HR = 1.19 (1.05 - 1.35)
logrank P = 0.0059
Probability
Time (months)
Expression
low
high
Number at risk
low 963 439 111 27 4
high 963 389 92 30 3
B
FBXL14
HR = 0.63 (0.53 - 0.75)
logrank P = 1.2e-07
Number at risk
low 577 270 94 35 5
high 568 276 45 11 1
C
FBXL3
HR = 0.59 (0.5 - 0.7)
logrank P = 3.8e-10
Number at risk
low 573 223 47 16 1
high 572 323 92 30 5
D
FBXO30
HR = 0.81 (0.68 - 0.95)
logrank P = 0.01
Number at risk
low 572 258 68 23 2
high 573 288 71 23 4

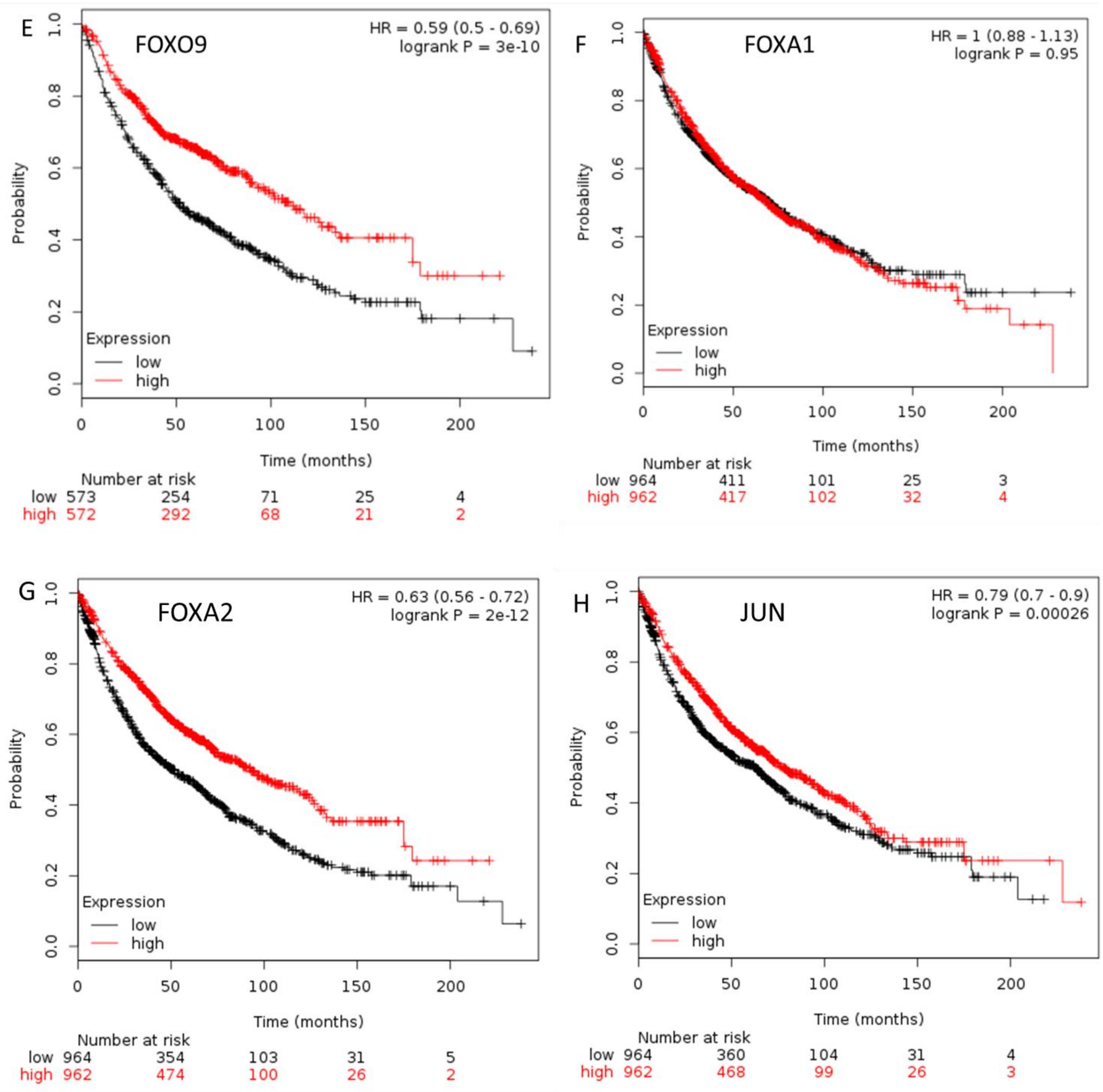

E
FOXO9
HR = 0.59 (0.5 - 0.69)
logrank P = 3e-10
Probability
Time (months)
Expression
low
high
Number at risk
low 573 254 71 25 4
high 572 292 68 21 2
F
FOXA1
HR = 1 (0.88 - 1.13)
logrank P = 0.95
Probability
Time (months)
Expression
low
high
Number at risk
low 964 411 101 25 3
high 962 417 102 32 4
G
FOXA2
HR = 0.63 (0.56 - 0.72)
logrank P = 2e-12
Probability
Time (months)
Expression
low
high
Number at risk
low 964 354 103 31 5
high 962 474 100 26 2
H
JUN
HR = 0.79 (0.7 - 0.9)
logrank P = 0.00026
Probability
Time (months)
Expression
low
high
Number at risk
low 964 360 104 31 4
high 962 468 99 26 3

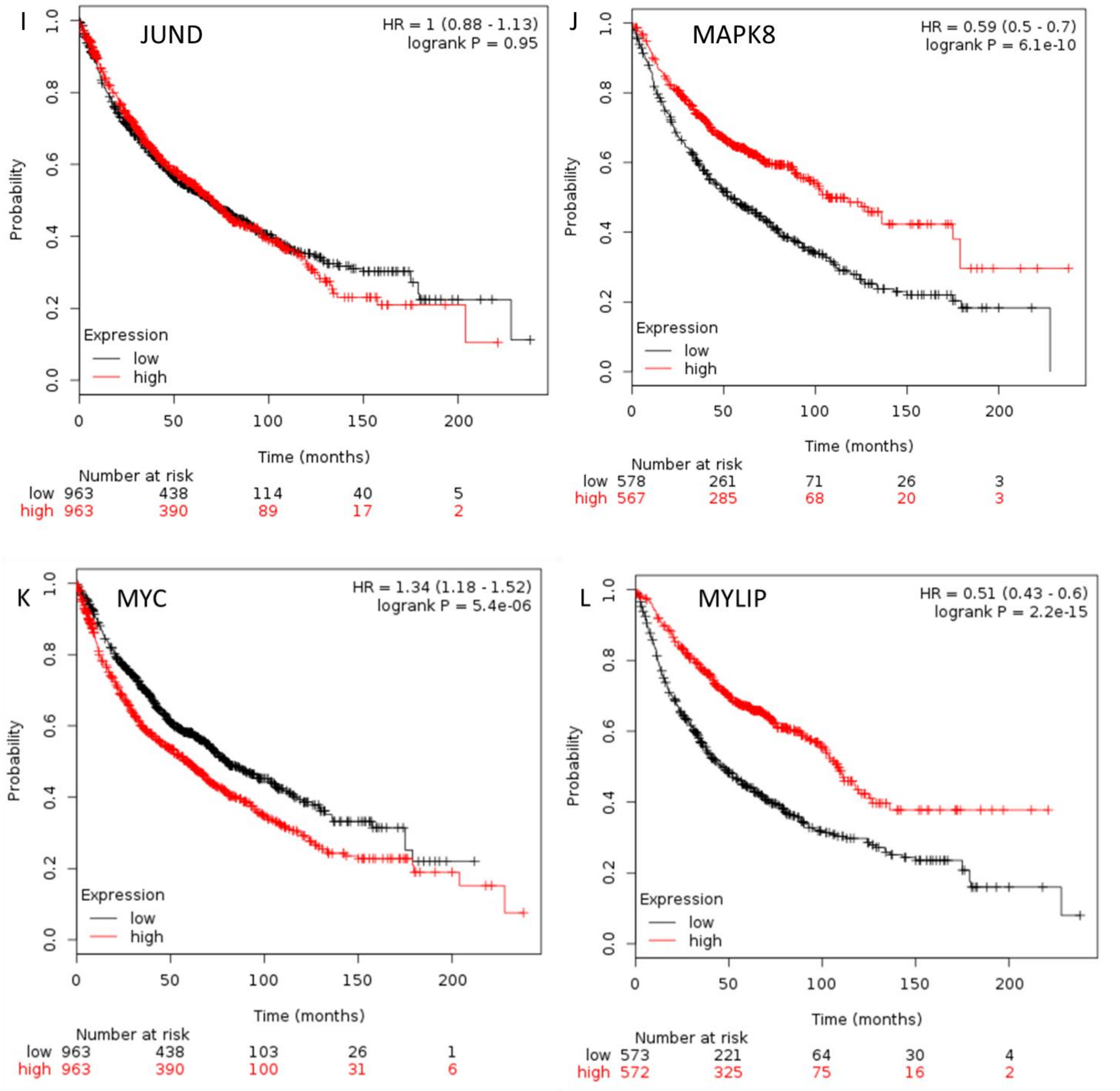
I
JUND
HR = 1 (0.88 - 1.13)
logrank P = 0.95
Probability
Time (months)
Expression
low
high
Number at risk
low 963 438 114 40 5
high 963 390 89 17 2
J
MAPK8
HR = 0.59 (0.5 - 0.7)
logrank P = 6.1e-10
Probability
Time (months)
Expression
low
high
Number at risk
low 578 261 71 26 3
high 567 285 68 20 3
K
MYC
HR = 1.34 (1.18 - 1.52)
logrank P = 5.4e-06
Probability
Time (months)
Expression
low
high
Number at risk
low 963 438 103 26 1
high 963 390 100 31 6
L
MYLIP
HR = 0.51 (0.43 - 0.6)
logrank P = 2.2e-15
Probability
Time (months)
Expression
low
high
Number at risk
low 573 221 64 30 4
high 572 325 75 16 2

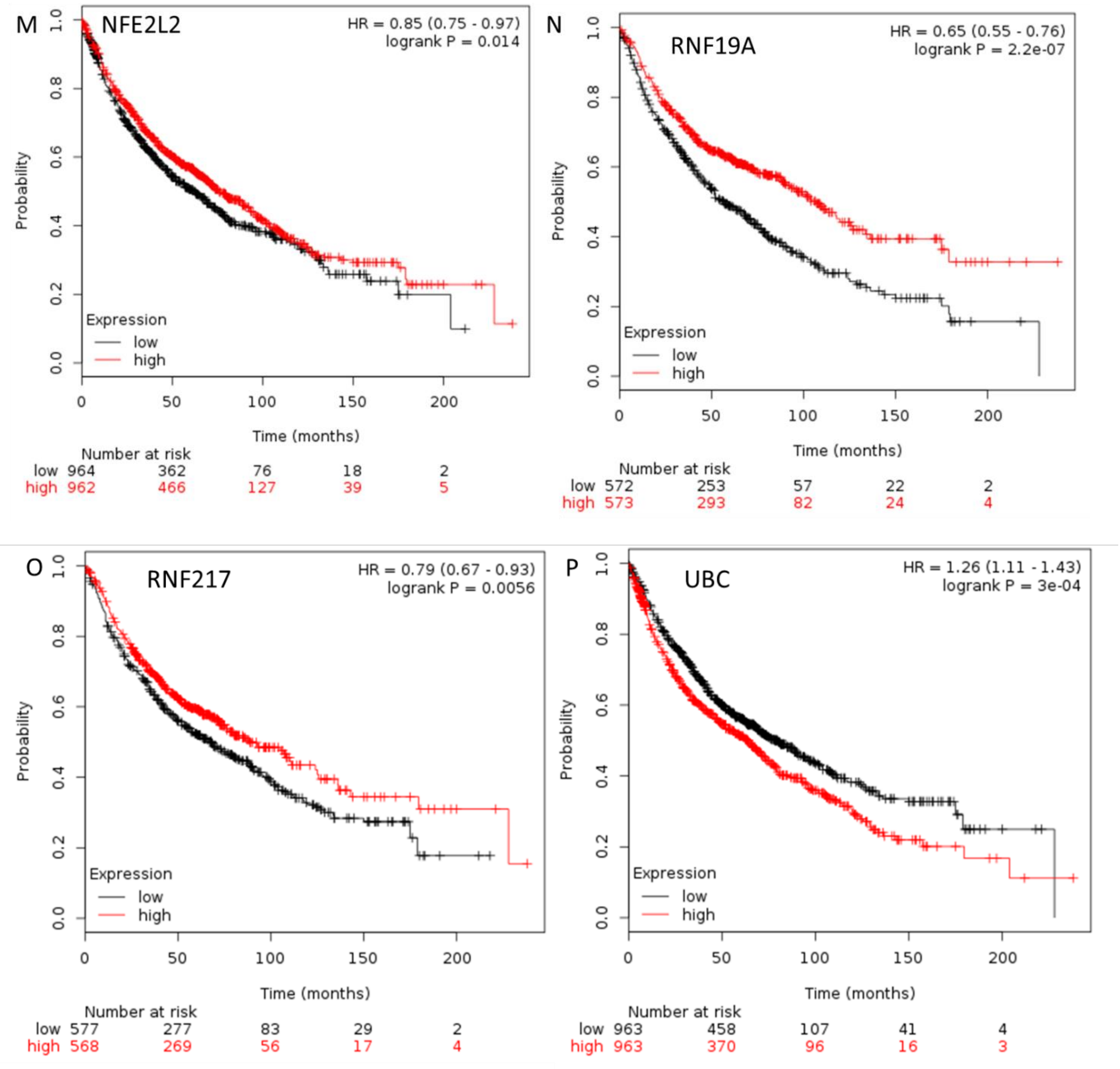

Figure 2: **Survival analysis using treatment biomarker genes.** A) CEBPB, B) FBXL14, C) FBXL3, D) FBXO30, E) FBXO9, F) FOXA1, G) FOXA2, H) JUN, I) JUND, J) MAPK8, K) MYC, L) MYLIP, M) NFE2L2, N) RNF19A, O) RNF217, and P) UBC. The red and black curve denote samples having gene expression value above or below threshold, which is median for each gene. The hazard ratio (HR) is computed as high-expression group vs low-expression group. X-axis is the timeline in months and Y-axis is the probability of survival of each subgroup. Also, below the X-axis, the number of samples for each subgroup are given at each time point. ***Conclusion:*** 2 biomarkers (FOXA1 and JUND) out of 16, do not have prognostic capability of differentiating between high-expression and low-expression groups of cancer patients, meaning hazard ratio is close to 1 (HR ≈ 1); 3 biomarkers (CEBPB, MYC and UBC) show that low-expression groups have higher chance of survival (HR > 1); and the remaining 11 biomakers show that high-expression groups have higher chance of survival (HR < 1).
